# Effects of continuous glucose monitoring on physical activity and diet in diabetes: a systematic review and meta-analysis

**DOI:** 10.1186/s12966-025-01870-0

**Published:** 2026-01-21

**Authors:** Mengsi Peng, Peng Shen, Kwang Joon Kim

**Affiliations:** 1 College of Pharmacy, Chonnam National University, 77 Yongbong-ro, Buk-gu, Gwangju, 61186 Korea, Republic of (South Korea); 2 School of Pharmaceutical Science, Wenzhou Medical University, Wenzhou, Zhejiang China

**Keywords:** Continuous glucose monitoring, Physical activity, Diet, Diabetes

## Abstract

**Background:**

Continuous glucose monitoring (CGM) may facilitate behavior modifications among individuals with diabetes. However, existing evidence regarding its effectiveness remains inconsistent. This study aimed to evaluate the effectiveness of CGM in improving physical activity and diet for people with diabetes using both qualitative and quantitative approaches.

**Methods:**

We searched PubMed, Cochrane, Embase, and Web of Science on October 13, 2024 to identify studies utilizing CGM in people of any age with any type of diabetes that reported outcomes related to physical activity and/or diet and were eligible for systematic review. Randomized controlled trials in which CGM was used as the main intervention and whose outcomes could be quantitatively synthesized were included in the meta-analysis.

**Results:**

The initial search identified 13,128 records, then 28 studies met the inclusion criteria for the systematic review. Of these, 10 and 8 studies were included in the meta-analysis of physical activity and diet, respectively. Compared with controls, CGM significantly increased daily physical activity time (MD: 16.21 min/day, 95% CI: 10.26 to 22.16; *P*<0.0001) (low-certainty evidence). In addition, CGM significantly reduced daily caloric intake (-70.81 kcal/day, -132.93 to -8.69; *P* = 0.03) (low-certainty evidence) and carbohydrate consumption (-19.88 g/day, -27.74 to -12.01; *P* < 0.00001) (low-certainty evidence) versus controls.

**Conclusions:**

CGM significantly increased daily physical activity time and reduced both caloric intake and carbohydrate consumption in adults with diabetes. Future high-quality, large-scale randomized controlled trials are warranted to confirm these associations and to clarify the mechanisms underlying CGM’s behavioral effects across diverse diabetes populations.

**Trial registration:**

International Prospective Register of Systematic Reviews (PROSPERO; CRD42024609764).

**Supplementary Information:**

The online version contains supplementary material available at 10.1186/s12966-025-01870-0.

## Background

Diabetes has emerged as one of the most pressing global public health challenges of the 21 st century, fueled by rising obesity rates, sedentary lifestyles, and unhealthy dietary patterns [1]. The International Diabetes Federation reported that the global number of individuals with diabetes reached 537 million in 2021, with projections estimating an increase to 783 million by 2045 if current trends continue [2]. Addressing this widespread chronic disease crisis requires scientifically grounded and effective management strategies to slow disease progression, prevent complications, and reduce diabetes-related mortality [1].

The biggest challenge in diabetes management is maintaining glycemic stability, as frequent or severe fluctuations in blood glucose not only accelerate the development and progression of diabetic complications but also substantially increase the risk of microvascular pathologies in patients with type 2 diabetes (T2D) [3]. Compared with conventional, invasive, and intermittent self-monitoring of blood glucose (SMBG), continuous glucose monitoring (CGM) technology uses subcutaneous microsensors to provide real-time tracking of interstitial glucose levels. This enables painless, continuous, and around-the-clock surveillance, offering multidimensional insights into glycemic variability [4]. Notably, CGM can visualize the dynamic interconnections between lifestyle factors (such as diet and physical activity) and glucose fluctuations with high temporal resolution [5]. Clinical evidence has shown that CGM facilitates tighter glycemic control relative to conventional SMBG [6, 7] by providing real-time glucose data, which allows people with diabetes and clinicians to make informed behavior or therapeutic adjustments to lower mean fasting and postprandial glucose levels [8]. These benefits have led to recent clinical guidelines endorsing CGM as a standard-of-care tool across various types of diabetes [9].

CGM’s visual feedback makes it a powerful facilitator for promoting proactive behavior adjustment. However, CGM is essentially an empowering tool whose true value lies in enhancing capacity for self-management of people with diabetes to reduce abnormal blood glucose fluctuations [10]. Physical activity and diet are critical for maintaining blood glucose homeostasis. Evidence indicates that engaging in at least 150 min of moderate-intensity aerobic exercise per week leads to measurable improvements in glycemic variability [11], whereas performing over one hour of daily moderate-to-vigorous intensity physical activity can reduce the risk of T2D by up to 74% [12]. Nutritional optimization strategies, such as replacing refined carbohydrates with low-glycemic index foods and adopting moderate carbohydrate restriction, have proven effective in stabilizing glycemic profiles and lowering cardiometabolic risk [11, 13]. CGM translates abstract metabolic fluctuations into concrete visual curves, which enhance individuals’ understanding of the relationships among diet, physical activity, and blood glucose. This, in turn, significantly increases their self-efficacy in making behavior change [14–17].

Despite the recognized benefits and clinical endorsement of CGM in diabetes management, most studies have focused on biochemical outcomes, with limited attention to its effects on lifestyle behaviors. One scoping review has addressed behavioral outcomes; however, it neither systematically synthesized the data nor rigorously assessed the quality of evidence [18]. To address this gap and enhance the robustness of existing evidence, we conducted this systematic review and meta-analysis quantifying the effects of CGM on physical activity and dietary intake in people with diabetes.

## Methods

This systematic review and meta-analysis followed the Preferred Reporting Items for Systematic Reviews and Meta-Analyses (PRISMA) reporting guideline [19]. The study protocol was registered with the International Prospective Register of Systematic Reviews (PROSPERO; CRD42024609764) on November 4, 2024. Data extraction was initiated only after protocol registration and was conducted in strict accordance with the predefined methods and outcomes specified in the PROSPERO record. 

### Data sources and search strategies

A comprehensive search was conducted in PubMed, Cochrane Library, Embase, and Web of Science on October 13, 2024, with no restriction on the start date. The research question was structured according to the PICO framework, and relevant Medical Subject Headings (MeSH) terms were identified, including diabetes mellitus, continuous glucose monitoring, exercise, and diet. To ensure comprehensive coverage of each concept, synonyms for each MeSH term were identified [20, 21]. A structured search strategy was developed by combining synonyms within each PICO element using the Boolean operator OR and linking different elements using AND. Further details are available in Supplementary File 1. Additionally, reference lists of previous meta-analyses and reviews were also tracked to identify relevant studies not captured by normal search strategies.

### Inclusion and exclusion criteria

Eligibility criteria for the systematic review were: (1) implementation of CGM as a primary intervention; (2) enrollment of individuals of any age with any type of clinically diagnosed diabetes mellitus; (3) reporting of outcomes related to physical activity and/or diet; and (4) no restrictions were placed on study design, including randomized controlled trials (RCTs), single-arm interventional studies, cohort studies, case-control studies, and single-group feasibility or acceptability studies.

Additional inclusion criteria for the meta-analysis were: (1) use of a RCT design; (2) studies in which the effects of CGM could be evaluated independently of other interventions; and (3) availability of extractable quantitative data on physical activity or diet, with sufficient methodological homogeneity to allow statistical pooling.

Exclusion criteria were: (1) non-English language publications; (2) preclinical or animal studies; (3) conference abstracts or unpublished manuscripts without full-text availability; and (4) non–peer-reviewed sources such as editorials, commentaries, or gray literature.

### Data screening and data extraction

After deduplication in EndNote, two reviewers independently screened titles and abstracts in duplicate. Studies meeting the eligibility criteria were subjected to full-text review. Discrepancies were resolved through consensus or consultation with a third qualified reviewer.

A structured data extraction protocol, adapted from a comparable systematic review methodology [18], was piloted and refined before use. Extracted fields included demographic and clinical characteristics, intervention details, outcomes, and CGM-specific information. Dual data extraction was performed from full texts and supplementary materials, with additional review of protocols and trial registries as needed. Missing data were labelled as “unclear,” and non-applicable fields as “NA.” Data were standardized by converting medians and interquartile ranges to means and standard deviations, calculating BMI and dropout rates from available data, and converting Hemoglobin A1c (HbA1c) values to percentages using the National Glycohemoglobin Standardization Program (NGSP) standard. Additionally, we extracted information on conflicts of interest and funding sources for all included studies, particularly noting any potential links to CGM manufacturers.

The study designated changes in physical activity as the primary outcome and changes in diet as the secondary outcome. For studies with long-term follow-up, data from the final time point were used. In studies with multiple control groups, data were merged when appropriate; if merging was not appropriate, the eligible subgroup(s) were selected for further analysis. Missing values extracted from figures were reconstructed using GetData Graph Digitizer 2.22. Physical activity outcomes were categorized as metabolic equivalent (MET) values, daily physical activity time (min/day), and daily moderate to vigorous physical activity (MVPA) time (min/day). Dietary data were stratified into caloric intake—calculated as [(carbohydrates + protein) × 4 + (total fat) × 9] (kcal/day) —and macronutrient intake (carbohydrates, protein, fat) in grams per day. For Carbohydrates Routinely Consumed (servings), values were converted to grams using the standard convention of approximately 15 g per serving [22]. Due to the lack of reported data on alcohol and dietary fiber, their contributions to total caloric intake could not be included in the calculation. In accordance with the Cochrane Handbook, all pre- and post-intervention data were converted into mean differences and standard deviations using standardized methods [23, 24].

### Quality and certainty assessment

The latest version of the Cochrane Risk of Bias 2 (RoB 2) tool was used to assess the risk of bias in RCTs included in the meta-analysis across five domains: randomization process, deviations from intended interventions, missing outcome data, measurement of the outcome, and selection of the reported result [25]. Each domain was rated as low risk, high risk, or some concerns based on responses to a series of signaling questions. An overall risk of bias judgment was derived automatically by the RoB 2 tool based on the information entered in each domain, with manual adjustments allowed based on study-specific context. Due to the inherent challenges of blinding in CGM interventions, downgrades related to lack of blinding were appropriately adjusted to “some concerns.”

The Grading of Recommendations Assessment, Development and Evaluation (GRADE) framework was utilized to evaluate the quality of evidence for each outcome [26, 27]. The assessment considered five key aspects: risk of bias, inconsistency, indirectness, imprecision, and publication bias. Each item was rated as not serious, serious, very serious, or extremely serious according to the GRADE handbook [28]. The evaluations were performed using the GRADEpro Guideline Development Tool (GDT), which automatically integrates the domain-specific ratings to generate an overall certainty of evidence categorized as high, moderate, low, or very low. The RoB 2 and GRADE assessments were independently conducted by two researchers and reviewed by a third to resolve any discrepancies.

### Statistical analysis

Statistical analysis was conducted using Review Manager (RevMan) version 5.4 to evaluate significant differences in physical activity and dietary changes between the CGM and control groups. As all outcome measures were continuous variables, the inverse variance method was used to calculate the mean difference (MD); point estimates were reported along with 95% confidence intervals (CIs). The threshold for statistical significance was regarded as *P* < 0.05. The choice of statistical model was based on the I² statistic. A random-effects model was utilized when I² exceeded 50%; otherwise, a fixed-effects model was used. The I² statistic quantifies the proportion of total variation across studies due to heterogeneity. Values above 25% are conventionally interpreted as indicating moderate heterogeneity. To explore potential sources of heterogeneity, a sensitivity analysis was conducted using the leave-one-out method. For outcomes with few studies or substantial heterogeneity, quantitative synthesis was conducted but interpreted cautiously.

In addition to the between-group meta-analysis, exploratory within-group (pre–post) analyses were conducted and are reported in the Supplementary Material. These results were not used to draw primary conclusions.

## Results

### Study selection and characteristics

**Fig. 1:**
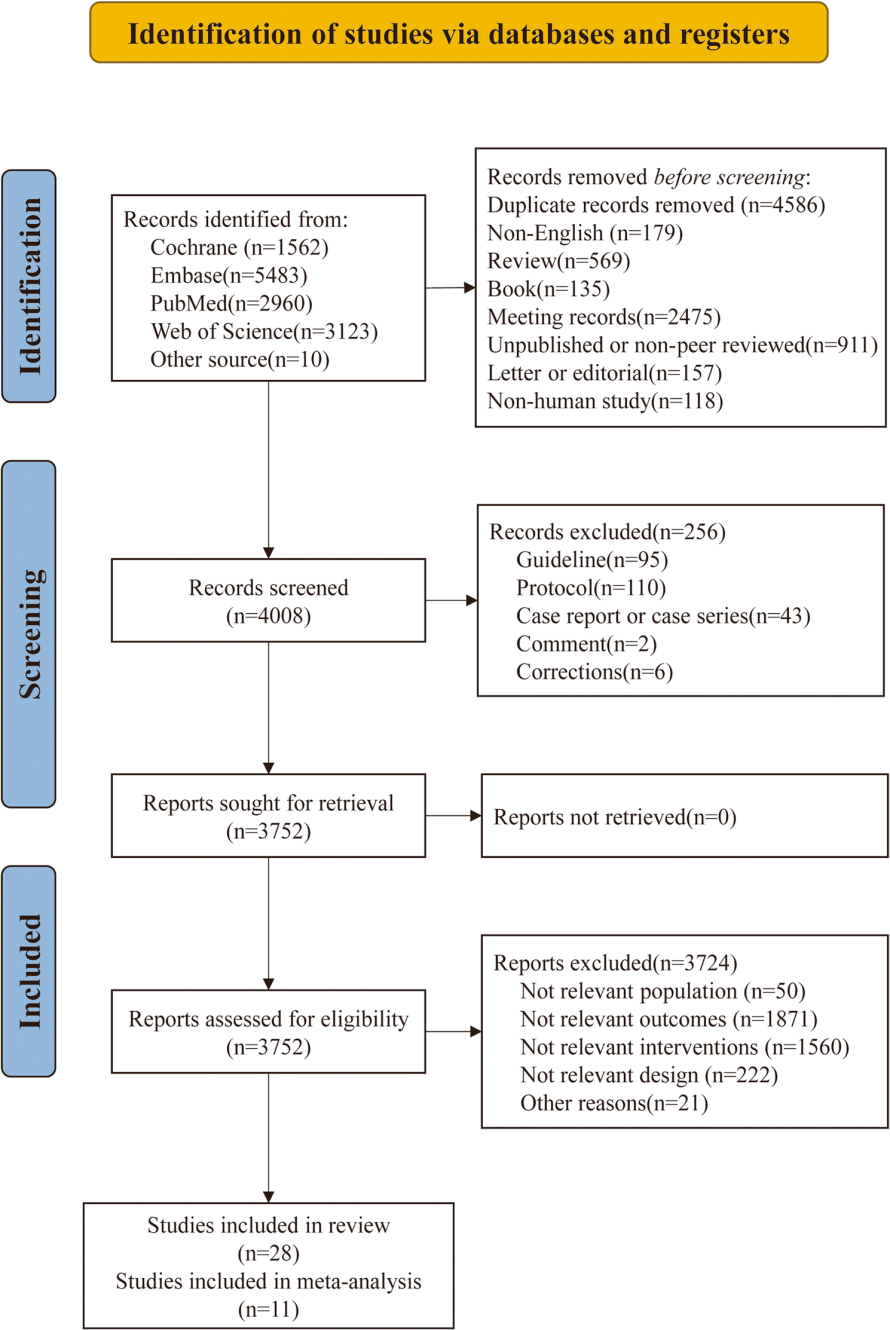
PRISMA flow diagram of study selection

The initial literature search identified 13,128 records. After deduplication (k = 4,586) and exclusion of studies that did not meet the predefined inclusion criteria (k = 4,800), 3,752 records proceeded to title and abstract screening. Full-text assessment was performed for 49 articles, leading to the exclusion of 21 studies; reasons are detailed in Supplementary Table 1. Of the 28 studies included in the systematic review, 16 RCTs were further assessed for eligibility in the meta-analysis. Three were excluded because CGM efficacy was not clearly evaluated [29–31], and two were excluded due to insufficient quantitative data despite attempts to obtain additional information from the authors [8, 32]. These five studies [8, 29–31], were retained in the systematic review and included in the qualitative synthesis (see Supplementary Table 2). Ultimately, 10 studies were included in the meta-analysis of physical activity, and 8 included in the analysis of dietary outcomes. The selection process is illustrated in Fig. 1.

The detailed characteristics of the included studies are presented in Table 1. The 28 studies consisted of 16 RCTs, 4 single-arm interventional trials, 3 single-group feasibility studies, 4 cohort studies, and 1 case-control study. Geographically, the majority of studies originated from the United States (k = 10), followed by South Korea, which contributed 3 RCTs. China, India, Canada, and Japan each contributed 2 studies; additional studies originated from Australia, Poland, Portugal, Sweden, and Saudi Arabia. Publication dates ranged from 2008 to 2024, with the earliest RCT reported by Allen et al. in March 2008 [32]. Notably, 68% (19/28) of all studies and 69% (11/16) of RCTs were published within the past 5 years. Among the 16 RCTs, 3 explicitly identified physical activity as the primary outcome [30, 32, 33]; only 1 of these was eligible for inclusion in the meta-analysis [33]. None of the included trials identified dietary outcomes as a primary endpoint.

**Table 1:** Characteristics of studies included in the systematic review and meta-analysis

Number	Author	Year of publication	Title	Country	Study design	Number of participants	Population	%female	Number of female	Mean age, y	Age range, y	BMI, kg/m2	Mean diabetes duration (year)
1	Ahn et al.	2023	Effectiveness of Non-Contact Dietary Coaching in Adults with Diabetes or Prediabetes Using a Continuous Glucose Monitoring Device: A Randomized Controlled Trial	Korea	RCT	50	PreD, T2D	62%	31	47±10	18-70	27.9±4.5	Unclear
2	Allen et al.	2008	Continuous glucose monitoring counseling improves physical activity behaviors of individuals with type 2 diabetes: A randomized clinical trial	USA	RCT	52	T2D	52%	27	57±13	30-84	35.0±5.9	8.4±6.2
3	Allen et al.	2011	A Continuous Glucose Monitoring and Problem-Solving Intervention to Change Physical Activity Behavior in Women with Type 2 Diabetes: A Pilot Study	USA	RCT(a pilot study)	29	T2D	100%	29	52±7	30-65	Unclear	6.7±5.2
4	Cox 1 et al.	2020	Glycemic excursion minimization in the management of type 2 diabetes: a novel intervention tested in a randomized clinical trial	USA	RCT	178	T2D	60%	106	58±12	30-80	34.9±6.2	5.5±3.1
5	Cox et al.	2021	Long-term follow-up of a randomized clinical trial comparing glycemic excursion minimization (GEM) to weight loss (WL) in the management of type 2 diabetes	USA	RCT	172	T2D	60%	103	58±12	30-80	34.9±6.2	5.5±3.1
6	Lee et al.	2022	FGM-based remote intervention for adults with type 1 diabetes: The FRIEND randomized clinical trial	Korea	RCT	36	T1D	53%	19	44±13	19-75	23.3±5.8	17.1±10.4
7	Taylor et al.	2019	Efficacy of Real-Time Continuous Glucose Monitoring to Improve Effects of a Prescriptive Lifestyle Intervention in Type 2 Diabetes: A Pilot Study	Australia	RCT(a pilot study)	20	T2D	50%	10	61± 8	20-75	35.5±3.1	Unclear
8	Yan et al.	2022	Real-Time Flash Glucose Monitoring Had Better Effects on Daily Glycemic Control Compared With Retrospective Flash Glucose Monitoring in Patients With Type 2 Diabetes on Premix Insulin Therapy	China	RCT	203	T2D	36%	73	61±10	43-82	25.0±3.2	Unclear
9	Yoo et al.	2008	Use of a real time continuous glucose monitoring system as a motivational device for poorly controlled type 2 diabetes	Korea	RCT	65	T2D	58%	38	55± 9	20-80	26.2±3.5	12.5±5.4
10	Kitazawa et al.	2024	Lifestyle Intervention With Smartphone App and isCGM for People at High Risk of Type 2 Diabetes: Randomized Trial	Japan	RCT	179	T2D	20%	36	48±8	20-80	26.5±3.5	Unclear
11	Cox 2 et al.	2020	Minimizing Glucose Excursions (GEM) With Continuous Glucose Monitoring in Type 2 Diabetes: A Randomized Clinical Trial	USA	RCT	30	T2D	63%	19	53±8	30-80	34.2±5.7	5.6±2.6
12	Bailey et al.	2016	Self-Monitoring Using Continuous Glucose Monitors with Real-Time Feedback Improves Exercise Adherence in Individuals with Impaired Blood Glucose: A Pilot Study	Canada	RCT(a pilot study)	13	PreD or T2D	71%	9	62±7	18-75	Unclear	Unclear
13#	Majewska et al.	2023	Flash glucose monitoring in gestational diabetes mellitus (FLAMINGO): a randomised controlled trial	Poland	RCT	100	GDM	100%	100	32±5	28-37	23.8±9.7	Unclear
14	Kytö et al.	2024	Periodic mobile application (eMOM) with self-tracking of glucose and lifestyle improves treatment of diet-controlled gestational diabetes without human guidance: a randomized controlled trial	Finland	RCT	148	GDM(24 to 28 weeks’ gestation)	100%	148	34±4	31-37	27.1±5.0	Unclear
15	Borel et al.	2024	Closed-Loop Insulin Therapy for People With Type 2 Diabetes Treated With an Insulin Pump: A 12-Week Multicenter, Open-Label Randomized, Controlled, Crossover Trial	France	Crossover RCT	17	T2D	35%	6	63±9	45-81	32.0±4.0	24.0±9.0
16	Nyström et al.	2024	Evaluation of Effects of Continuous Glucose Monitoring on Physical Activity Habits and Blood Lipid Levels in Persons With Type 1 Diabetes Managed With Multiple Daily Insulin Injections: An Analysis Based on the GOLD Randomized Trial (GOLD 8)	Sweden	Crossover RCT	143	T1D with MDI	44%	63	45±13	19-77	Unclear	22.2±11.8
17	Polonsky et al.	2023	The AH-HA! Project: Transforming Group Diabetes Self-Management Education Through the Addition of Flash Glucose Monitoring	USA	Single-arm interventional study	35	T2D	26%	9	56±11	21-75	Unclear	2.6 ± 1.6
18	Ida et al.	2020	Effects of Flash Glucose Monitoring on Dietary Variety, Physical Activity, and Self-Care Behaviors in Patients with Diabetes	Japan	Single-arm interventional study	90T1D-42 T2D-48	T1D or T2D	52%	47	57±9	20-75	Unclear	Unclear
19	Mohan et al.	2016	Use of Retrospective Continuous Glucose Monitoring for Optimizing Management of Type 2 Diabetes in India	India	Single-arm interventional study	181	T2D	34%	62	54±10	19-70	27.1±4.1	14.6±8.1
20	Ribeiro et al.	2021	Impact of blinded retrospective continuous glucose monitoring on clinical decision making and glycemic control in persons with type 2 diabetes on insulin therapy	Portugal	Single-arm interventional study	102	T2D without adequate glycaemic control	50%	51	58±1	55-61	31.0±0.5	16.9±0.8
21	Allen et al.	2009	Feasibility and acceptability of continuous glucose monitoring and accelerometer technology in exercising individuals with type 2 diabetes	USA	Single-group feasibility study	9	T2D	22%	2	56±9	38-74	32.5±4.2	3.7±3.7
22	Fritschi et al.	2010	Continuous glucose monitoring: the experience of women with type 2 diabetes	USA	Single-group feasibility study	35	T2D	100%	35	53±7	40-65	Unclear	6.1±6.0
23	Litchman et al.	2022	Continuous Glucose Monitoring Plus an Online Peer Support Community Reinforces Healthy Behaviors in Hispanic Adults With Type 2 Diabetes	USA	Single-group feasibility study	26	T2D	62%	16	55±10	25-85	Unclear	6.1±6.2
24	Al Hayek et al.	2021	Effectiveness of the freestyle libre 2 flash glucose monitoring system on diabetes-self-management practices and glycemic parameters among patients with type 1 diabetes using insulin pump	Saudi Arabia	Prospective cohort study	47	T1D	45%	21	16±6	13-21	22.5±2.5	51.1%≤5 yrs, 48.9%>5 yrs
25	Zahedani et al.	2023	Digital health application integrating wearable data and behavioral patterns improves metabolic health	USA	Prospective cohort study	2217	Glucose in normal range, preD or T2D	51%	1131	49±12	25-73	NA	NA
26	Wong et al.	2023	Use of Personal Continuous Glucose Monitoring (CGM) with Support in People with Type 1 and 2 Diabetes Treated with Insulin in the Outpatient Clinic: A Single-Center Retrospective Cohort Study	China	Retrospective cohort study	180	T1D or T2D	42%	76	59±13	33-85	Unclear	18.5±8.6
27	Jabbour et al.	2021	Continuous Blood Glucose Monitoring Increases Vigorous Physical Activity Levels and Is Associated With Reduced Hypoglycemia Avoidance Behavior In Youth With Type 1 Diabetes	Canada	Retrospective cohort study	61	Youth with T1D	46%	28	14±2	5-17	20.8	≥1
28	Kesavadev et al.	2017	Assessing the Therapeutic Utility of Professional Continuous Glucose Monitoring in Type 2 Diabetes Across Various Therapies: A Retrospective Evaluation	India	Retrospective case-control study	592	T2D	27%	160	54±12	30-78	Unclear	13.5±6.8

Number	Author	HbA1c eligibility criteria (NGSP, %)	HbA1c eligibility criteria (IFCC, mmol/mol)	Baseline HbA1c (%)	Medication use	Study duration	Intervention components	Control group	Dropout rate (%)	Primary and secondary outcomes	Physical activity-related measurements	Diet-related measurements
1	Ahn et al.	diabetes: HbA1c of ≥6.5%, prediabetes: 5.7<HbA1c<6.4%	HbA1c≥39mmol/mol	6.9±1.1	Medications use to control blood glucose (BG) levels	4w	Unblind CGM and nurse-led diet coaching (dietary education, diet feedback, individual coaching and group coaching) for 4 weeks	Unblind CGM and conventional care (education on PA and compliance with the medications)	10%	Diet, depression, insomnia, BMI, waist and thigh circumferences, HbA1c level, apolipoprotein B (ApoB)/apolipoprotein A (ApoA) ratio, and the HOMA-IR score.	NA	Weight efficacy lifestyle short-form questionnaire
2	Allen et al.	HbA1c >7.5%	HbA1c>58mmol/mol	8.6±1.2	Insulin use no	8w	Unblinded CGM(three days at week 1) and retrospective CGM-based guidance on activity and individualized education	Individualized diabetes education for 90 minutes.	12%	PA, BP, HbA1c, and BMI.	Self-efficacy for Exercise Behavior (SEBS)Physical activity levels: accelerometers (a product of movement frequency and intensity)	NA
3	Allen et al.	HbA1c > 7.0%	HbA1c>53mmol/mol	8.6±1.3	Insulin use no	12w	CGM counseling intervention on PA (week 1, glucose responses to events, benefits of PA), and problem-solving counseling (week 4, discuss the specific barriers in implementing the PA prescription).	CGM counseling intervention on PA (week 1, glucose responses to events, benefits of PA), attention-control diabetes education (week 4, without PA and diet).	7%	PA, diet, DPSI, depression, HbA1c, weight, and BP.	SEBSPhysical activity levels: accelerometers (a product of movement frequency and intensity)	A subscale of the Summary of the Diabetes Self-Care Activities
4	Cox 1 et al.	HbA1c≥6.8%	HbA1c≥51mmol/mol	8.3±1.3	Insulin use if clinically indicated	13w	Unblinded CGM and education focused on glycaemic excursion minimization (GEM).	Standard weight loss (WL) education on diet and physical activity.	11%	HbA1c, lipids, weight, PA, diet, depression, diabetes empowerment, diabetes distress, MES.	A blinded activity monitor (steps/day, Hours active)	Automated Self-Administered 24-hour Dietary Assessment Tool (ASA24)
5	Cox et al.	HbA1c≥6.8%	HbA1c≥51mmol/mol	8.3±1.3	Insulin use no	13m follow-up	Unblinded CGM and four 90-minute group sessions focused on reducing postnutrient glucose excursions by dietary and PA guidance in three months. No maintenance program was employed between postassessment and 13-month follow-up.	Four 90-minute group sessions focused on reducing postnutrient glucose excursions by education on diet and PA.	10%	HbA1c, lipids, cardiovascular risk, diabetes distress, diabetes empowerment, GMSS, depression, frequency of SMBG, MES, diet.	NA	Carbohydrates Routinely Consumed (servings)
6	Lee et al.	HbA1c ≥7.0%	HbA1c≥53mmol/mol	8.9±1.6	Insulin use yes	12w	Unblinded CGM and retrospective CGM-based individual guidance on diet, medication, and PA.	Unblinded CGM without intervention	6%	HbA1c, CGM metrics, DTSQ, depression, anxiety, total daily doses of insulin, daily scan times, diet, PA, BMI, waist circumference.	Number of exercise per weekHours of exercise per week	Number of meals per dayNumber of snacks per day
7	Taylor et al.	5.9%<HbA1c<6.9%	41mmol/mol<HbA1c<52mmol/mol	6.9±0.8	Insulin use if clinically indicated	12w	Unblinded CGM with prospective CGM-based guidance on diet and activity; diet, glucose, and PA tracking; diet and PA prescription.	Blinded CGM (without feedback) and diet, glucose, and PA tracking; diet and PA prescription.	0%	HbA1c, fasting glucose, glycaemic variability, lipids, MES, weight, body composition, BP, PA, MAGE.	Seven consecutive days of ambulatory accelerometer monitoring: percentage of time spent in sedentary behavior daily, percentage of time spent in moderate/vigorous activity daily.	NA
8	Yan et al.	HbA1c ≥ 7.0%	HbA1c ≥ 53mmol/mol	7.5±1.2	Use premix insulin	13w	Unblinded CGM and CGM-based guidance on medication changes; diet and PA tracking; individual education and study log (record diet and exercise for all days).	Blinded CGM (with feedback after wear), diet and PA tracking, individual education and study log (record diet and exercise for all days).	15%	TIR, TBR, TAR, MBG, SDBG, CV, hourly mean blood glucose, C-peptide, HbA1c, PA, diet, and insulin dose per day.﻿	Daily exercise time(min/day)	Diet log, number of meals
9	Yoo et al.	8.0<HbA1c<10%	64mmol/mol<HbA1c<86mmol/mol	8.9±0.9	Insulin use mixed	13w	Unblinded CGM and CGM-based guidance on diet and activity and individual education.	Individual education and glucometer-based advice.	12%	HbA1c, fasting blood glucose, postprandial 2 h blood glucose, lipid profiles, weight, waist circumference, BMI, diet, and PA.﻿	Exercise time per week (min/week)	3-day food records analyzed by Can-Pro 3.0
10	Kitazawa et al.	5.6%<HbA1c<6.4%	38mmol/mol<HbA1c<46mmol/mol	5.8±0.3	Unclear	12w	Unblinded CGM and Health 2Sync mobile app (tracking food records, PA records and diabetes-related information; providing guidance on diet and activity based on BG data from isCGM)	No lifestyle modification information and no use of health care related smartphone applications	6%	HbA1c, CGM metrics, body weight, BMI, waist circumference, BP, PA, diet, MAGE, CONGA.	Japanese version of the International Physical Activity Questionnaire (IPAQ) short form	Brief Diet History Questionnaire
11	Cox 2 et al.	HbA1c≥7.0%	HbA1c≥53mmol/mol	8.9±1.6	No nondiabetic medications that could affect BG control, medications use to control BG levels	12w	Unblind CGM with 4 group sessions of glycemic excursion minimization, which provided a manual and a diary that focused on diet, activity, and management of hypoglycemia.	Routine care (RC)	0%	HbA1c, BMI, depression, MES, diabetes knowledge, diabetes distress, diabetes empowerment, GMSS, SMBG frequency, PA, diet.	A blinded activity monitor (Fitbit Charge 2), average daily active minutes	ASA24
12	Bailey et al.	PreD: 5.7%<HbA1c<6.4%T2D: HbA1c>6.4%	PreD: 39mmol/mol<HbA1c<46mmol/molT2D: HbA1c>46mmol/mol	Unclear	Unclear	8w	Unblinded CGM, guidance on how to self-monitor exercise and BG, to goal set, and to use CGM to observe how exercise influences BG and exercise program two times per week for 8 weeks.	Standard care and exercise program two times per week for 8 weeks.	23%	Self-monitoring and goal setting, self-efficacy, PA, SF-36, BMI, waist circumference, 6-min walk test, BG self-monitoring frequency	7-Day Physical Activity Recall Questionnaire (7-day PAR)	NA
13#	Majewska et al.	(1) fasting plasma glucose 5.11–6.94 mmol/L(2) 1-h glucose concentration≥10.0mmol/L(3) 2-h glucose concentration 8.5–11.06 mmol/L	(1) fasting plasma glucose 5.11–6.94 mmol/L(2) 1-h glucose concentration≥10.0mmol/L(3) 2-h glucose concentration 8.5–11.06 mmol/L	(1) OGTT-fasting glycaemia (mmol/L): 4.94±0.57(2) 1 h OGTT (mmol/L): 9.76±1.65(3) 2 h OGTT (mmol/L): 7.71±1.77	Unclear	12-16w	FGM system and guidance on glycaemic control, diet recommendations, and physical activity.	SMBG and guidance on on glycaemic control, diet recommendations, and physical activity.	1%	Fasting and 1 h-postprandial glu cose concentrations (after breakfast, lunch, and dinner), qualification to insulin therapy and dosage, diet, PA, gestational weight gain, weeks of gestation, route of birth, newborn weight and neonatal hypoglycaemic events.	Daily footsteps	Eating Assessment Test (EAT)
14	Kytö et al.	(1) fasting plasma glucose≥5.1mmol/L(2) 1-h glucose concentration≥10.0mmol/L(3) 2-h glucose concentration≥8.5mmol/L	(1) fasting plasma glucose≥5.1mmol/L(2) 1-h glucose concentration≥10.0mmol/L(3) 2-h glucose concentration≥8.5mmol/L	(1) OGTT-fasting glycaemia (mmol/l): 5.3±0.09(2) 1 h OGTT (mmol/l): 8.7±0.4(3) 2 h OGTT (mmol/l): 7.14±0.4	No medication use	7-13w	Unblinded CGM with standard care in combination with use of the periodic mobile application (eMOM) with wearable sensors, an activity tracker, and a food diary 1 week/month until delivery	Standard care	22%	Change in fasting plasma glucose from baseline to 35 to 37 weeks’ gestation, capillary glucose, weight gain, diet, PA, pregnancy complications, and neonatal outcomes (macrosomia).﻿	A blind accelerometer	Semiquantitative 142-item food-frequency questionnaire
15	Borel et al.	HbA1c<10%	HbA1c<86mmol/mol	7.9±0.9	Insulin use yes	13w	Use a hybrid closed-loop device, which was composed of an Accu-Chek Insight insulin pump and the DEXCOMG6 CGM system.	Use usual insulin pump therapy (CSII) and CGM.	0%	TIR, other CGM metrics (glycemic variability, TAR, TBR, GMI), PA, sleep, DTSQ, treatment safety, satisfaction with the therapy.	Mean daily physical activity (METs) by 1-week actimetry	NA
16	Nyström et al.	HbA1c≥7.5%	HbA1c≥58 mmol/mol	8.7±0.8	Multiple daily insulin injections	16m(6m intervention, 4m wash-out period)	Unblinded CGM and guidance on insulin dosing and bolus correction, food choices, and the potential effect of PA on glucose control.	SMBG and guidance on insulin dosing and bolus correction, food choices, and the potential effect of PA on glucose control.	18%	PA, blood lipids, apolipoproteins, hsCRP, creatinine, HbA1c, BP and weight	IPAQ	NA
17	Polonsky et al.	HbA1c ≥8.0%	HbA1c >64 mmol/mol	mean glucose: 10.2±3.4mmol/L	Unclear	8w	Five weekly group sessions focused on medicines, meals, movement, mood, and minutes/timing and training on FGM placement, wear, and maintenance.	NA	0%	CGM metrics, diabetes distress, WHO-5, diabetes self-care behaviors (medication adherence, healthful eating, PA)	Diabetes self-care behaviors: times of more than 30 min PA per week	Diabetes self-care behaviors-Healthful eating
18	Ida et al.	7%≤HbA1c<10%	53mmol/mol<HbA1c<86mmol/mol	7.7±1.2	Biguanides (64%), dipeptidyl peptidase-4 inhibitors (37%), andsodium-glucosecotransporter2inhibitors(37%)	12w	Unblinded CGM with BG checking intervals less than 8 h, repeatedly guidance on diet and exercise therapies.	NA	0%	DVS, PA, SDSCA, DTSQ, HbA1c, MAGE, CGM metrics, body weight, daily insulin dose	The Japanese version of the IPAQ	The Dietary Variety Score (DVS)
19	Mohan et al.	8.0%<HbA1c<10.0%	64mmol/mol<HbA1c<86mmol/mol	8.6±1.1	Insulin use if clinically indicated	12w	Visit five times to get information about the application, downloading data, reviewing the report of the CGM, and receiving CGM-based guidance on therapy modifications (diet and PA).	NA	18%	HbA1C, changes to treatment regimen, questionnaires concerned diabetes control and self-management.	Questionnaires concerned diabetes control and self-management.	Questionnaires concerned diabetes control and self-management.
20	Ribeiro et al.	HbA1c >7.5%	HbA1c >58 mmol/mol	9.4±0.1	Metformin(72%), DPP4 inhibitors（26%). Mixed insulin analogues (58%), long-acting insulin analogue (34%), and fast-acting insulin analogue (32%)	52w	A 7-day blinded rCGM every four months for one year, a diary to record the type and timing of food intake, PA and medication, SMBG values, and diabetes-related events and extra consultations for nursing assistance, nutrition or education if necessary.	NA	12%	HbA1c, waist circumference, BMI, CGM metrics, hypolycaemia, episodes, GHQ, DTSQ, education and pharmacological therapeutic changes, open qualitative question.﻿	NA	Qualitative exploratory questionnaire (nutrition consultation component)
21	Allen et al.	Unclear	Unclear	6.4±7.0	Insulin use no, metformin (55.6%), a glitazone (44.4%) and a sulfonylurea (44.4%), long-acting diabetes medications(100%)	3d	Phase 1—Instructed participants about wearing the CGM monitor and activity monitors for 72 hours. Then, CGM data were downloaded and reviewed with each participant. Phase 2—A one-hour, tape recorded focus group interview moderated by the interventionist.	NA	22%	PA, glucose levels, BMI, HbA1c, CGM metrics, participants’ experiences and perceptions, diabetes medications.	An activity monitor (uniaxial accelerometer): type, frenquency, duration, intensity.	NA
22	Fritschi et al.	Unclear	Unclear	7.2±1.8	Oral medications(74.3%), insulin injections(5.7%), oral medications and insulin injections(5.7%)	3d	Wore a CGM for 3 days, then semistructured interviews were conducted to capture the self-described experience of wearing a CGM.	NA	0%	Semistructured Interviews	Open-ended questions related to PA	Open-ended questions related to diet
23	Litchman et al.	Unclear	Unclear	Unclear	Insulin use no	12w	A 12-week combined CGM and Online Peer Support Community (OPSC) intervention, then followed semistructured interviews.	NA	0%	Semistructured interviews	Open-ended questions related to PA	Open-ended questions related to diet
24	Al Hayek et al.	Unclear	Unclear	8.3±2.5	Received insulin for at least 12 months before inclusion	12w	Received comprehensive learning and written instructions about CGM to improve self-care management ability.	NA	0%	CGM metrics, HbA1c, TDDI, glycemic variability, frequency of glucose monitoring, DSMQ.﻿	Diabetes Self Management Questionnaire (DSMQ)	DSMQ
25	Zahedani et al.	NA	NA	NA	NA	12w	Wore CGM over 28 days to capture glucose patterns and get personalized recommendations based on CGM data and records of food intake, PA, and body weight via a smartphone app. Users could interact with the app for an additional 2 months without CGM.	NA	0%	TIR, GMI, mean number of hyperglycemic events per day, weight loss, Heart rate, PA, diet	Active logging of daily physical activity	Active logging of all meals
26	Wong et al.	Unclear	Unclear	8.4±1.2	4 or more insulin injections daily (majority), metformin(87.7%), SGLT2 inhibitor(52.3%)	12-20w	Set up and educate on CGM use; 10–14 days of CGM + food/insulin/activity log book; written recommendations based on CGM report.	Usual care: instructed by doctors to modify lifestyle or diabetes medications according to HbA1c during regular outpatient visits.	0%	HbA1c, percentage of people achieving HbA1c reduction, Pharmacological interventions change, Nonpharmacological interventions (patient education, hypoglycemic management, exercise management, and dietary advice), GMSS	Exercise management	Logbooks with dietary information
27	Jabbour et al.	Unclear	Unclear	7.7±1.6	Unclear	NA	A self-administered questionnaire included age, sex, height, weight, duration of diabetes, hypoglycemia episodes, fear of hypoglycemia scores, insulin therapy (pump vs. injection), and blood glucose monitoring (CGM vs. BGM) methods, and PA levels.	NA	0%	The frequency of hypoglycemia episodes, CHFS, CHMS, insulin therapy (pump vs. injection), BGM methods	Cycle 2 of the Canadian Health Measures Survey (Cycle 2 of CHMS)	NA
28	Kesavadev et al.	HbA1c<7.0%	HbA1c<53 mmol/mol	7.5±1.4	Insulin with OHA(91%), one or more OHAs without insulin(7%), GLP-1RAs(2%)	6m	Routine care and treatment advice based on pCGM report and personal diet and exercise analysis during usual clinical visits and virtual consultations through Diabetes Tele-Management System (DTMS).	Routine care and treatment advice during usual clinical visits and virtual consultations through DTMS.	Unclear	HbA1c, SMBG frequency, treatment options, Recommendations prescribed to patients﻿	A log book recording physical activity	A log book recording diet

Number	Author	Outcomes relate to physical activity (PA) and diet	Outcomes relate to PA and diet-simplied	Main outcomes	Adverse event	CGM type	Sensor brand/model	CGM blinding	Channel	Frequency	Attached area	Conflict of interest & funding
1	Ahn et al.	Eating self-efficacy improved greater for women in the experimental group compared with those in the control group, and the change in eating self-efficacy was negatively related to insomnia.	Experimental group: Eating self-efficacy (for women) ⬆，the change in eating self-efficacy was negatively related to insomnia.	After the intervention, the thigh circumference in men significantly differed between the two groups. For women, participants in the experimental group showed greater improvement in eating self-efficacy compared with those in the control group. Insomnia was negatively related to the change in eating self-efficacy and increase in thigh circumference.	Unclear	isCGM	Abbott Freestyle Libre	Unblinded	A user device or smartphone	Continous	The back of the upper arm	Funding: NoConflict of Interest: NoCGM brand support: No
2	Allen et al.	Participants in the experimental group had higher self-efficacy scores for sticking to activity than the control group at 8 weeks; their light/sedentary activity minutes decreased significantly and moderate activity minutes increased significantly.	Experimental group: higher self-efficacy scores for sticking to activity, light/sedentary activity minutes⬇, moderate activity minutes⬆	Participants receiving the intervention had higher self-efficacy scores than the control group for sticking to activity/resisting relapse at 8 weeks, indicating more confidence in maintaining a PA program. Intervention group participants light/sedentary activity minutes decreased significantly, moderate activity minutes increased significantly , and, HbA1c and BMI decreased significantly.	Unclear	Unclear	Medtronic Minimed	Unblinded	Unclear	Once(three days at week 1)	Unclear	Funding: YesConflict of Interest: NoCGM brand support: Yes
3	Allen et al.	Participants in the CGM plus problem-solving group had significantly greater dietary adherence and moderate activity minutes than those in the CGM plus education group, although not statistically significant.	Experimental group: greater dietary adherence and moderate activity minutes.	Continuous glucose monitoring plus problem-solving group participants had significantly greater problem-solving skills and had greater, although not statistically significant, dietary ad herence, moderate activity minutes, weight loss, and higher intervention satisfaction pre- to post-intervention than did participants in the continuous glucose monitoring plus education group.	Unclear	Unclear	Brand and model unspecifed	Unclear	Unclear	Unclear	Unclear	Funding: NoConflict of Interest: NoCGM brand support: No
4	Cox 1 et al.	Participants in the experimental group significantly increased steps/day, and active hours and reduced carbohydrate intake and calories/day.﻿	Experimental group: steps/day⬆, and active hours⬆, carbohydrate intake⬇, calories/day⬇.	While WL reduced BMI, GEM demonstrated a greater reduction in HbA1c, BMI, carbohydrate intake, BG response to a glucose challenge, and cardiovascular risk. Only GEM participants significantly improved diabetes empowerment, diabetes distress, depressive symptoms, steps/day, and active hours and reduced calories/day.	Unclear	Unclear	Dexcom G5	Unblinded	DexCom Clarity app	Intermittent (one 7-day sensor at each treatment session, and another sensor at 8 weeks after the last treatment.)	Unclear	Funding: YesConflict of Interest: NoCGM brand support: Yes
5	Cox et al.	Participants in the experimental group continued to reduce their carbohydrate ingestion in the follow-up period.	Experimental group: carbohydrate ingestion⬇.	Pre to follow- up within- group comparisons indicated WL participants sustained improvement in BMI . GEM participants continued to benefit in their HbA1c, BMI, high- density lipoprotein, reduction of carbohydrate ingestion, self- monitoring of blood glucose satisfaction and frequency, diabetes knowledge, diabetes empowerment, and both diabetes distress emotional and regimen subscales. Forty- two percent and 52% of WL and GEM participants, respectively, were classified as responders (individuals whose A1c dropped by at least −0.5%, with a mean HbA1c reduction of −1.2% and −1.5%.	No adverse events	Unclear	Dexcom G5	Unblinded	DexCom Clarity app	Intermittent (one 7-day sensor at each treatment session, and another sensor at 8 weeks after the last treatment.)	Unclear	Funding: YesConflict of Interest: NoCGM brand support: Yes
6	Lee et al.	No improvements in diet or exercise were found.	No improvements in diet or exercise were found.	FGM use significantly improved HbA1c levels by −1.4% and −0.8% in both groups with and without remote intervention, respectively. However, the intervention group showed significant increases in time with glucose in the range of 70–180 mg/dL and significant decreases in time with hyperglycemia and meanglucose, but the control group did not.Moreover, the TIR, time with hyperglycemia >250 mg/dL, and coefficient of variation were significantly improved in the intervention group compared to the control group.In particular, the CGM metrics improved gradually as the remote intervention was repeated.Furthermore, the intervention group reported higher treatment satisfaction.No improvements in diet, exercise, body mass index, and waist circumference were found.	Unclear	isCGM	Abbott Freestyle Libre	Unblinded	LibreView	Continous	Unclear	Funding: YesConflict of Interest: NoCGM brand support: No
7	Taylor et al.	Percentage time spent in sedentary behavior and percentage time spent in moderate/vigorous activity were similar in both groups at 12 weeks.	Percentage time spent in sedentary behavior and percentage time spent in moderate/vigorous activity were similar in both groups at 12 weeks.	Both groups experienced reductions in body weight, HbA1c, fasting blood glucose, LDL-C and triglycerides; with no differential effect between groups. At week 12, GV indices were consistently lower by at least sixfold in rtCGM compared to control, although there was insufficient power to achieve statistical significance. Overall, there was an approximately 40% greater reduction in blood glucose-lowering medication (MeS) in rtCGM compared to control.	Unclear	rtCGM	Medtronic Guardian Connect	Unblinded	iPod device Bluetooth connected to the CGM	Continous	Unclear	Funding: YesConflict of Interest: NoCGM brand support: No
8	Yan et al.	Participants in real-time FGMs increased daily exercise time compared with the retrospective FGM group.	Experimental group:daily exercise time⬆	Time in range increased significantly after 3 months in the real-time FGM group compared with the retrospective FGM group.HbA1c decreased in both groups.Real-time FGMs increased daily exercise time compared with the retrospective group.	Unclear	Ex: rtCGMCo: pCGM	Ex: Abbott Freestyle LibreCo: Abbott Freestyle Libre Pro	Ex: UnblindedCo: blinded	Unclear	Intermittent (14 days before week1, day31-45, day76-90 )	The back of the left upper arm	Funding: YesConflict of Interest: NoCGM brand support: Yes
9	Yoo et al.	In the rtCGM group, there was a significant reduction in total caloric intake. The total exercise time per week was significantly increased in the rtCGM group compared with the SMBG group.	Experimental group:total caloric intake⬇, total exercise time per week⬆	The HbA1c of the rtCGM group was significantly reduced after 12 weeks compared with the SMBG group. In the rtCGM group, there was a significant reduction in total daily caloric intake, weight, body mass index (BMI), and postprandial glucose level, and a significant increase in total exercise time per week after 3 months.	No reports of skin reactions or clinically significant hypoglycemic events.	rtCGM	Medtronic MiniMed	Unblinded	Unclear	Intermittent (once a month for 3 days)	Unclear	Funding: YesConflict of Interest: NoCGM brand support: Yes
10	Kitazawa et al.	Carbohydrate intake was significantly reduced in the intervention group compared with the control group, but the change in total energy intake and PA did not differ between groups. Decreases in carbohydrate and fat intakes and increases in protein intake did contribute to weight loss.	Experimental group: carbohydrate intake⬇	Intervention with a smartphone app and intermittently scanned continuous glucose monitoring increased glycemic control accompanied by decreased carbohydrate intake and weight loss. Further trials are needed to confirm whether these interventions can reduce incident type 2 diabetes.	Unclear	isCGM	Abbott Freestyle Libre	Unblinded	Linked to smartphone app	Intermittent(every two weeks for 14 days)	Unclear	Funding: YesConflict of Interest: YesCGM brand support: Yes
11	Cox 2 et al.	Participants in the experimental group reduced carbohydrate consumption relative to RC, but no difference in PA was found between groups. And the calories burned and minutes of daily moderate to vigorous activity correlated with change in HbA1c.	Experimental group: carbohydrate consumption⬇，the calories burned and minutes of daily moderate to vigorous activity correlated with change in HbA1c.	GEM participants significantly improved HbA1c compared with RC group. Additionally, GEM reduced the need for diabetes medication, reduced carbohydrate consumption, and improved diabetes knowledge, quality of life and diabetes distress, and trended to more empowerment without increasing dietary fat, lipids, or hypoglycemia.GEM did not lead to an increase in caloric, fat, or pro tein consumption nor a worsening in lipids relative to RC.	The experimental group reported level 1 symptomatic hypoglycemia(n=11).	rtCGM for interventon	Dexcom Platinum G4 for assessment, Dexcom G5 for intervention	Unblinded for interventon, blind for assessment	Unclear	Intermittent (rtCGM at week2, week3,week5, week8; isCGM at week1 and 3-month follow-up)	Unclear	Funding: YesConflict of Interest: NoCGM brand support: Yes
12	Bailey et al.	Participants in the experimental group had greater increases in exercise levels and a significantly greater percentage of attendance compared with the control group.	Experimental group: exercise levels⬆, higher percentage of attendance.	Repeated-measures analysis of variance revealed significant Condition · Time interactions for self monitoring, goal setting, and self-efficacy to self-monitor, such that the SM condition showed greater increases in these outcomes immediately after the program and at the 1-month follow-up compared with the control condition. The self-monitoring condition had higher program attendance rates, and a greater proportion of participants reregistered for additional exercise programs compared with the control condition. Participants in both conditions experienced improvements in health-related quality of life, waist circumference, and fitness.On average, participants in the self-monitoring condition attended a significantly greater percentage of classes (96.88%) than those in the control condition(84.70%). The odds of reregistering were 12.50 times higher if participants were in the SM condition compared with the control condition.	Unclear	rtCGM	Medtronic MiniMed	Unblinded	Electronic monitor	Intermittent (week 1, week 4, and week 7 for 5 days )	Unclear	Funding: YesConflict of Interest: NoCGM brand support: No
13#	Majewska et al.	Compared to the SMBG group, pregnant women in isCGM group modify their diet habits more frequently, leading to strict glycaemic control that consequently lower the incidence of fetal macrosomia.	Experimental group: frequency of diet habits modifications⬆, better glycaemic control, incidence of fetal macrosomia⬇.	There was no signifcant diference in mean glycaemia between the groups compared to the control group, the study group signifcantly reduced their fasting and postprandial glycaemia during the frst 4 weeks following GDM diagnosis, with no signifcant diference in progression to insulin therapy. Incidence of fetal macrosomia was signifcantly higher in SMBG as compared to FGM group.The study group more frequently modified their diet habits, when compared to the control group. Compared to the SMBG group, isCGM system could detect masked hypoglycaemic events in pregnancy, help preganants modify their diet habit, led to strict glycaemic control, that consequently decreased mean fasting and postprandial glycaemias at follow up visits, and then lower the incidence of fetal macrosomia.	Neonatal hypoglycaemic events were reported in the experimental group(n=4), and control group(n=10).	isCGM	Abbott Freestyle Libre	Unclear	Unclear	Once(first 4 weeks)	Unclear	Funding: NoConflict of Interest: NoCGM brand support: No
14	Kytö et al.	Participants in the experimental group increased intake of vegetables, decreased sedentary behavior, and increased light physical activity when compared with the control group. Adherence to eMOM was correlated with lower intake of energy and carbohydrates and longer duration of the daily PA.	Experimental group: intake of vegetables⬆, light physical activity⬆, sedentary behavior⬇.	The intervention arm showed a lower mean change in fasting plasma glucose than the control arm and lower capillary fasting glucose levels. The inter vention arm also increased their intake of vegetables, decreased their sedentary behavior, and increased light physical activity when compared with the control arm. In addition, gestational weight gain was lower, and there were less newborns with macrosomia in the intervention arm. Adherence to eMOM was high, and the usage correlated with lower maternal fasting and postprandial glucose levels, weight gain, intake of energy and carbohydrates, and longer duration of the daily physical activity. There were no significant between-arm differences in terms of pregnancy complications.	Unclear	Unclear	Medtronic	Unblinded	eMOM system	Intermittent(1 week/month until delivery)	Unclear	Funding: YesConflict of Interest: YesCGM brand support: Yes
15	Borel et al.	The mean daily physical activity did not differ between groups.	The mean daily physical activity did not differ between groups.	TIR in creased to 76.0% during the closed-loop condition vs. 61.0% during the CSII + CGM condition; mean difference was 15.0 percentage points. A decrease in time above range, in glucose management indicator, in glucose variability, and an increase in daily insulin dose. Actimetric sleep analysis showed an improvement in sleep fragmentation during closed-loop treatment. Closed-loop therapy improved glycemic control more than did CSII + CGM in people with type 2 diabetes.The mean daily physical activity did not differ between conditions.	Severe adverse event (hyperglycemia) related to study(n=1), adverse event related to study(n=11).	rtCGM	Dexcom G6	Unblinded	Diabeloop application, YourLoops web platform	Continous	Unclear	Funding: YesConflict of Interest: YesCGM brand support: Yes
16	Nyström et al.	No significant changes existed in PA during CGM and SMBG.	No significant changes existed in PA during CGM and SMBG.	No significant changes existed in physical activity, lipid-lowering medication, low-density lipoprotein, high-density lipoprotein, triglycerides, total cholesterol, apolipoprotein A1, apolipoprotein B1, or hsCRP, during CGM and SMBG. Only HbA1c differed significantly by 4.2 mmol/mol between SMBG and CGM treatment.	Unclear	Unclear	DexCom G4	Unblinded for about six months, blinded during two last weeks of the six months	Unclear	Continous	Unclear	Funding: YesConflict of Interest: YesCGM brand support: Yes
17	Polonsky et al.	Participants reported improvements in healthy eating and physical activity, although the latter did not reach statistical significance.	Participants reported improvements in healthy eating and physical activity, although the latter did not reach statistical significance.	There was a significant gain in percentage TIR 70–180 mg/dL from baseline to month 3, and a parallel drop-in percentage TAR>180 mg/dL from 44% to 25%.Overall well-being rose significantly, whereas diabetes distress showed a nonsignificant drop.Participants reported improvements in healthy eating and physical activity, although the latter did not reach statistical significance.	Unclear	isCGM	Abbott Libre Pro for assessmentAbbott FreeStyle Libre 2 for intervention	Unblinded throughout 8-week intervention period, blinded at baseline and month 3 assessment	A shared viewing portal (LibreView)	Continous	Unclear	Funding: YesConflict of Interest: YesCGM brand support: Yes
18	Ida et al.	In patients with T2D, there was an increase in moderate/high category scores for IPAQ. No change was observed in IPAQ in patients with T1D.	In patients with T2D, there was an increase in moderate/high category scores for IPAQ. No change was observed in IPAQ in patients with T1D.	In patients with T2D, there was an increase in moderate/high category scores for IPAQ and for treatment satisfaction reported via DTSQ. Furthermore, in patients with T2D, the glycemic excursion index improved significantly and HbA1c decreased significantly. Standard deviation and mean amplitude of glycemic excursions significantly decreased in patients with T1D.	Unclear	isCGM	FreeStyle Libre	Unblinded	Unclear	Continous	The upper arm	Funding: NoConflict of Interest: NoCGM brand support: No
19	Mohan et al.	67.6% and 48.6% of patients changed their exercise habit and diet during the study, respectively.	67.6% and 48.6% of patients changed their exercise habit and diet during the study, respectively.	Most subjects (91.2%) had >1 therapy change after review of the first iPro2 test. Mean A1C decreased from 8.6% at baseline to 8.0% at month 3 (p<0.001). Questionnaire results from patients and HCPs indicated that both groups viewed the iPro2 studies and results as acceptable and useful.At least one therapy change was made in 142 (95.9%) of the completers. Among these 142, the mean A1C value decreased by 0.69±1.10 percentage points. Among the 6 (4.1%) completers who did not make a therapy change, the mean A1C value increased by 0.28±0.88 percentage points. The most frequent change was the addition o f insulin; many patients also changed their treatment regimens with respect to oral medications, diet, and exercise.	No serious adverse device effects.	Unclear	iPro2, Medtronic	Blind	iPro2 System	Continous	Unclear	Funding: YesConflict of Interest: YesCGM brand support: Yes
20	Ribeiro et al.	The first CGM resulted in 50% of participants being referred for a nutrition consultation, and another 7% discussed specific food counseling during CGM review. By 12 months, 29% discussed aspects of their dietary plan during CGM review.﻿	The first CGM resulted in 50% of participants being referred for a nutrition consultation, and another 7% discussed specific food counseling during CGM review. By 12 months, 29% discussed aspects of their dietary plan during CGM review.﻿	Nocturnal exposure to hyperglycaemia decreased over the study period, while nocturnal hypoglycaemia showed a downward trend from baseline to 12 months. At baseline, CGM analysis prompted therapeutic adjustments in 99% of participants; at 12 months, this remained true for 84%. The first CGM led to nutrition consultations for 50% of participants and dietary counseling for 7%, whereas by 12 months, none were referred to nutrition consultations and 29% discussed dietary management during CGM review.	Unclear	pCGM	MiniMedTM Medtronic	Blind	iPro 2 rCGM system	Intermittent(a 7-day blinded rtCGM everyfour months for one year)	Unclear	Funding: YesConflict of Interest: NoCGM brand support: Yes
21	Allen et al.	CGMS feedback reinforced diet and exercise programs compared to formal diabetes education.	CGMS feedback reinforced diet and exercise programs compared to formal diabetes education.	Compared to formal diabetes education, visual data from the glucose monitoring technology were perceived as more relevant to participants’ particular, everyday experiences with exercise, diet and stress. Participants reported a reinforced commitment to their exercise and diet regimens after using Continuous Glucose Monitoring System.	Pinched skin when bending (n=1), sweaty and irritated skin under monitor (n=4)	Unclear	Medtronic Minimed	Blind	A communication device for downloading monitor data to a personal computer	Continous for 3 days	Unclear	Funding: YesConflict of Interest: NoCGM brand support: Yes
22	Fritschi et al.	Use of CGM heightened awareness of how activity and foods affect blood glucose levels, and the majority of participants would like to change their eating and exercise behaviors.	Use of CGM heightened awareness of how activity and foods affect blood glucose levels, and the majority of participants would like to change their eating and exercise behaviors.	After viewing the results, most women were surprised by the magnitude and frequency of blood glucose excursions. They immediately examined their behaviors during the time they wore the CGM. Independent problem-solving skills became apparent as they attempted to identify reasons for hyperglycemia by retracing food intake, physical activity, and stress experiences during the period of CGM. Most important, the majority of women stated they were interested in changing their diabetes-related self-care behaviors, especially eating and exercise behaviors, after reviewing their CGM	Unclear	Unclear	Medtronic MiniMed CGMS® Gold sensor	Blind	A software program	Continous for 3 days	Under the skin of the abdomen	Funding: YesConflict of Interest: NoCGM brand support: Yes
23	Litchman et al.	CGM helped participants recognize what types, portions, or combinations of foods raised their glucose levels and how their glucose levels reacted to being active.	CGM helped participants recognize what types, portions, or combinations of foods raised their glucose levels and how their glucose levels reacted to being active.	When combined, CGM and OPSC interventions appear to create a positive feedback loop to reinforce and optimize healthy behaviors for diabetes self-management in individuals with type 2 diabetes who are not on insulin.	Unclear	pCGM	Unclear	Blind	Unclear	Ccontinous	Unclear	Funding: YesConflict of Interest: YesCGM brand support: Yes
24	Al Hayek et al.	Participants got significant improvements in the dietary control and physical activity at 12 weeks compared to baseline.	Participants got significant improvements in the dietary control and physical activity at 12 weeks compared to baseline.	At baseline, mean HbA1c was 8.3%, decreasing to 7.9% at 12 weeks. Glucose monitoring frequency increased from 2.4/day with BGM to 8.2/day after using FSL2. Significant improvements were observed in all DSMQ subscales, including glucose management, dietary control, physical activity, health care use, and self-care.	Unclear	isCGM	Abbott Freestyle Libre 2	Unblinded	Free mobile applications (FreeStyle Libre Link and FreeStyle Libre LinkUp) and virtual software (LibreView)	Continous	Upper arm (back-side)	Funding: NoConflict of Interest: NoCGM brand support: No
25	Zahedani et al.	Participants, especially healthy and prediabetic subgroups, significantly increased daily physical activity, decreased caloric, carbohydrate, and sugar intake, and increased protein and fiber consumption during the study.	Daily physical activity⬆, caloric⬇, carbohydrate⬇, sugar⬇, increased protein⬆, fiber⬆.	Here we report significant improvements in hyperglycemia, glucose variability, and hypoglycemia, particularly in those who were not diabetic at baseline. Body weight decreased in all groups, especially those who were overweight or obese. Healthy eating habits improved significantly, with reduced daily caloric intake and carbohydrate-to-calorie ratio and increased intake of protein, fiber, and healthy fats relative to calories.	Unclear	Unclear	Freestyle Libre, Abbott	Unclear	January AI app	Continous for 28d	Unclear	Funding: YesConflict of Interest: YesCGM brand support: No
26	Wong et al.	77.9% of T2D patients agreed or strongly agreed that CGM helped them understand how diet and activity affect them. 54 (60.0%) and 3 (3.3%) people in the CGM group had dietary advice and exercise management, respectively.	77.9% of T2D patients agreed or strongly agreed that CGM helped them understand how diet and activity affect them. 54 (60.0%) and 3 (3.3%) people in the CGM group had dietary advice and exercise management, respectively.	CGM use led to greater reductions in mean HbA1c compared with control overall and in T2D, but not in T1D. In the CGM group, 69% had additional pharmacological adjustments, 60% received dietary advice, and 13.3% had insulin doses corrected for carbohydrate intake. A 10–14-day CGM with professional support was effective for improving glucose control in insulin-treated outpatients.	Unclear	rtCGM and isCGM	Dexcom G6 and Freestyle Libre	Unclear	Unclear	Continous	Unclear	Funding: NoConflict of Interest: NoCGM brand support: No
27	Jabbour et al.	There were significant differences in the components of PA profile between youth using CGM and those using BGM, and CGM use may be associated with increased vigorous PA among T1D youth. Those with higher hypoglycemia fear survey behavior scores engaged in more vigorous PA and had fewer hypoglycemia episodes.	There were significant differences in the components of PA profile between youth using CGM and those using BGM, and CGM use may be associated with increased vigorous PA among T1D youth. Those with higher hypoglycemia fear survey behavior scores engaged in more vigorous PA and had fewer hypoglycemia episodes.	No significant differences were found in hypoglycemia episodes, CHFS scores, or PA components between youth using injections versus pumps. However, differences emerged based on glucose monitoring method (CGM vs. blood glucose meters). Higher vigorous physical activity (VPA) was associated with higher CHFS behavior scores, which in turn correlated negatively with hypoglycemia episodes over the past 12 months. CGM use may be linked to increased VPA among youth with T1D, and those with higher CHFS behavior scores engaged in more VPA and experienced fewer hypoglycemia episodes.	Unclear	Unclear	Unclear	Unclear	Unclear	Unclear	Unclear	Funding: NoConflict of Interest: NoCGM brand support: No
28	Kesavadev et al.	Detailed analysis of the pCGM data led to appropriate changes in diet and exercise in almost all patients.	Detailed analysis of the pCGM data led to appropriate changes in diet and exercise in almost all patients.	pCGM revealed that the predominant pattern of hyperglycemia was postprandial while previously unknown hypoglycemia was found in 38% of the patients; over half of the cases of hypoglycemia were nocturnal. The mean A1C of the P-CGM group dropped from 7.5 ± 1.4% at baseline vs. 7.0±0.9% at 6 months. The frequency of performing self-monitoring of blood glucose (SMBG) was also found to be significantly increased in these patients from the baseline. Meanwhile, no significant improvement in A1C was noted in the control group during the same time frame and frequency of SMBG remained almost unchanged.	Unclear	pCGM	iPro2, Medtronic	Blind	Unclear	Continous	Unclear	Funding: NoConflict of Interest: YesCGM brand support: No

Values are presented as mean ± standard deviation#Data checked against the corrected version; no differences relevant to this review were identified

*Abbreviations*: *BMI* Body Mass Index, *HbA1c* Hemoglobin A1c (glycated hemoglobin), *NGSP* National Glycohemoglobin Standardization Program, *IFCC* International Federation of Clinical Chemistry and Laboratory Medicine, *PA* Physical Activity, *CGM* Continuous Glucose Monitoring, *RCT* randomized controlled trial, *PreD* prediabetes, *T2D* type 2 diabetes, *BG* Blood Glucose, *w* week, *HOMA-IR* Homeostatic Model Assessment for Insulin Resistance, *NA* not applicable, *isCGM* intermittently scanned Continuous Glucose Monitoring, *BP* blood pressure, *SEBS* Self-efficacy for Exercise Behavior Scale, *DPSI* Diabetes Problem-Solving Inventory, *GEM* glycemic excursion minimization, *WL* weight loss, *m* month, *GMSS* Glucose Monitoring Satisfaction Survey, *SMBG* Self-Monitoring of Blood Glucose, *MES* Medication Effect Score, *ASA24* Automated Self-Administered 24-hour Dietary Assessment Tool, *DTSQ* Diabetes Treatment Satisfaction Questionnaire, *T1D* type 1 diabetes, *TIR* time in range, *MAGE* mean amplitude of glucose excursion, *LDL-C* Low-Density Lipoprotein Cholesterol, *GV* Glycemic Variability, *rtCGM* real-time Continuous Glucose Monitoring, *TBR* Time Below Range, *TAR* Time Above Range, *MBG* Mean Blood Glucose, *SDBG* Standard Deviation of Blood Glucose, *pCGM* professional Continuous Glucose Monitoring, *CONGA* continuous overlapping net glycemic action, *IPAQ* International Physical Activity Questionnaire, *RC *Routine care, *SF-36* The Short Form 36 Health Survey, *7-day PAR* 7-Day Physical Activity Recall Questionnaire, *GDM* gestational diabetes mellitus, *OGTT* Oral Glucose Tolerance Test, *EAT* Eating Assessment Test, *eMOM* periodic mobile application, *CSII* usual insulin pump therapy, *GMI* Glucose Management Indicator, *METs* Metabolic Equivalents, *MDI* Multiple Daily Injections, *hsCRP* high-sensitivity C-reactive protein, *WHO-5* World Health Organization-Five Well-Being Index, *h* hour, *DVS* Dietary Variety Score, *SDSCA* the Summary of Diabetes Self-Care Activities, *GHQ* Global Health Questionnaire, *d* day, *OPSC* Online Peer Support Community, *TDDI* total daily dose of insulin, *DSMQ* diabetes self-management questionnaire, *BGM* Blood Glucose Monitoring, *SGLT2inhibitor* Sodium-Glucose Cotransporter 2 inhibitor, *CHMS* Canadian Health Measures Survey, *CHFS* Children’s Hypoglycemia Fear Survey, *OHA* oral hypoglycemic agents, *GLP-1Ras* glucagon-like peptide-1 receptor agonists, *DTMS* diabetes tele-management system

Adverse event reporting was limited, with only 25% (7/28) of the included studies providing relevant data. Three studies reported no adverse events [16, 34, 35]. The remaining 4 studies reported a total of 25 cases of hypoglycemia [17], 1 case of hyperglycemia [31], 1 case of skin pinching during activity [14], 4 cases of sweating and inflammation beneath the device [14], and 11 other study-related adverse events [31].

Out of 28 included studies, 16 were funded by CGM manufacturers, 9 declared author conflicts of interest, and the remaining studies reported no conflicts or did not report this information.

### Qualitative synthesis of the results

#### Participants

The total number of participants was 4,418, including 1,435 RCT enrollees. Female participants accounted for 2,454 (56%) of the overall sample and 817 (57%) of RCT participants, with 4 trials exclusively recruiting females [8, 30, 36, 37]. The age of participants spanned 5–85 years, with majority representation in the 50–60 year stratum. Two studies included children, comprising a total of 90 participants [38, 39]. Mean BMI values varied between 20.8 and 35.5 kg/m² across studies. Among 28 studies, 22 included people with T2D [14, 16, 17, 29–32, 34–36, 40–51]; 2 focused on gestational diabetes [8, 37]; 6 papers had people with type 1 diabetes (T1D) [33, 38, 39, 42, 49, 52]; and 3 studies included people with prediabetes [40, 51, 53]. Diabetes duration across the included studies ranged from 1 to 24 years, with a median of 8.4 years (interquartile range: 5.6–16.9 years). Baseline HbA1c values varied between 6.4% and 9.4%. Notably, some participants continued insulin or oral hypoglycemic therapy during the trial periods.

#### Interventions

Study durations varied widely, ranging from 3 days to 16 months, with a median duration of 12 weeks (interquartile range: 11–13 weeks). All RCTs incorporated CGM with additional self-management guidance/education focusing on diet (k = 11) [8–34, 41, 44, 48, 50, 52], physical activity (k = 13) [16, 17, 30, 32–34, 40, 41, 44, 48, 50, 52], or medication (k = 2) [50, 52], delivered through individual or group sessions. Most RCTs employed non-blinded CGM (k = 13) to develop feedback-driven guidance. Some studies specifically examined the synergistic effects of combining CGM with other interventions [29–31], but these were not included in the meta-analysis because the independent effect of CGM could not be isolated (Supplementary Table 2). Control arms included education/counseling (k = 7) [16, 30, 32–34, 41], routine care (k = 4) [17, 29, 37, 40], or blinded CGM (k = 2) [48, 50]. The single-arm trials [35, 42, 46, 47] and cohort studies [38, 39, 49, 51] also included interventions of CGM training and health education. In addition, 3 single-group feasibility studies employed semi-structured interviews after using CGM for 3 days or 12 weeks [14, 36, 45].

#### Outcomes

Among 28 studies, 25 reported physical activity outcomes. Objective measurement via accelerometers or activity monitors was employed in 7 studies [14, 17, 30, 32, 34, 37, 41], whereas self-reported data through participant diaries or questionnaires were used in 20 studies; some studies employed both objective and self-reported measurement methods. Among these, 3 studies utilized the International Physical Activity Questionnaire (IPAQ) [33, 42, 44], 2 applied the Self-Efficacy for Exercise Behavior Scale (SEBS) [30, 32], and 8 studies involved participant-recorded exercise logs [16, 31, 43, 46, 49–52]. Additional scales included the Diabetes Self-Management Questionnaire (DSMQ) [38], Cycle 2 of the Canadian Health Measures Survey (CHMS) [39] and the 7-day Physical Activity Recall Questionnaire (7-day PAR) [40].

Diet-related outcomes were reported in 21 of the 28 studies. Nearly all dietary intake assessments were based on participant-completed diaries or questionnaires. One study, however, evaluated dietary changes indirectly through documentation of education and pharmacological therapeutic adjustments, reporting the proportion of participants who received nutrition consultations or discussed dietary plans during CGM reviews [47]. Methods included simple diet diaries or meal logs (k = 8) [16, 34, 43, 46, 49–52], the Automated Self-Administered 24-hour Dietary Assessment Tool (ASA24) (k = 2) [17, 41], and open-ended questions (k = 2) [36, 45]. Validated scales, such as the Weight Efficacy Lifestyle Questionnaire (short form) [29], the diet subscale of the Summary of Diabetes Self-Care Activities [30], the Eating Assessment Test (EAT) [8], the Dietary Variety Score (DVS) [42], and the Diabetes Self-Management Questionnaire (DSMQ) [38], as well as validated questionnaires, including the Brief Diet History Questionnaire [44], and a 142-item semiquantitative food-frequency questionnaire [37], were also employed to evaluate dietary behavior.

#### Findings

CGM interventions were broadly associated with improvements in daily physical activity [14, 16, 32, 35, 37–40, 42, 43, 49–51], including increases in light [32, 37], moderate [32, 42], and vigorous activity levels [42], greater daily step counts [41], and better adherence to exercise programs [14, 32, 40, 49]. Regarding dietary behaviors, CGM use ehchanced dietary self-efficacy, adherence to recommended meal plans, and awareness of how different foods, portion sizes, and food combinations affected blood glucose levels [14, 29, 30, 35, 36, 38, 40, 45, 47, 49]. Several studies also documented reductions in carbohydrate [17, 34, 37, 41, 44, 51] and overall caloric intake [16, 41, 51], alongside higher consumption of protein [51], fiber [51], and vegetables [37]—collectively indicating improved dietary regulation. However, some studies reported no statistically significant changes in physical activity [17, 30, 31, 33, 44, 46, 48, 52] or diet [30, 52], particularly when CGM was implemented without accompanying problem-solving strategies or individualized behavioral counseling [30, 48, 52]. Notably, two single-group feasibility studies demonstrated that even short-term exposure to CGM (e.g., three days) increased participants’ health awareness and motivation for self-management [14, 36]. Overall, the qualitative synthesis suggests that CGM can support improvements in dietary behavior and physical activity, primarily by enhancing awareness, self-efficacy, and motivation. Nonetheless, the magnitude of these effects varies across intervention characteristics and populations.

### Effectiveness of CGM on physical activity (meta-analysis)

**Fig. 2:**
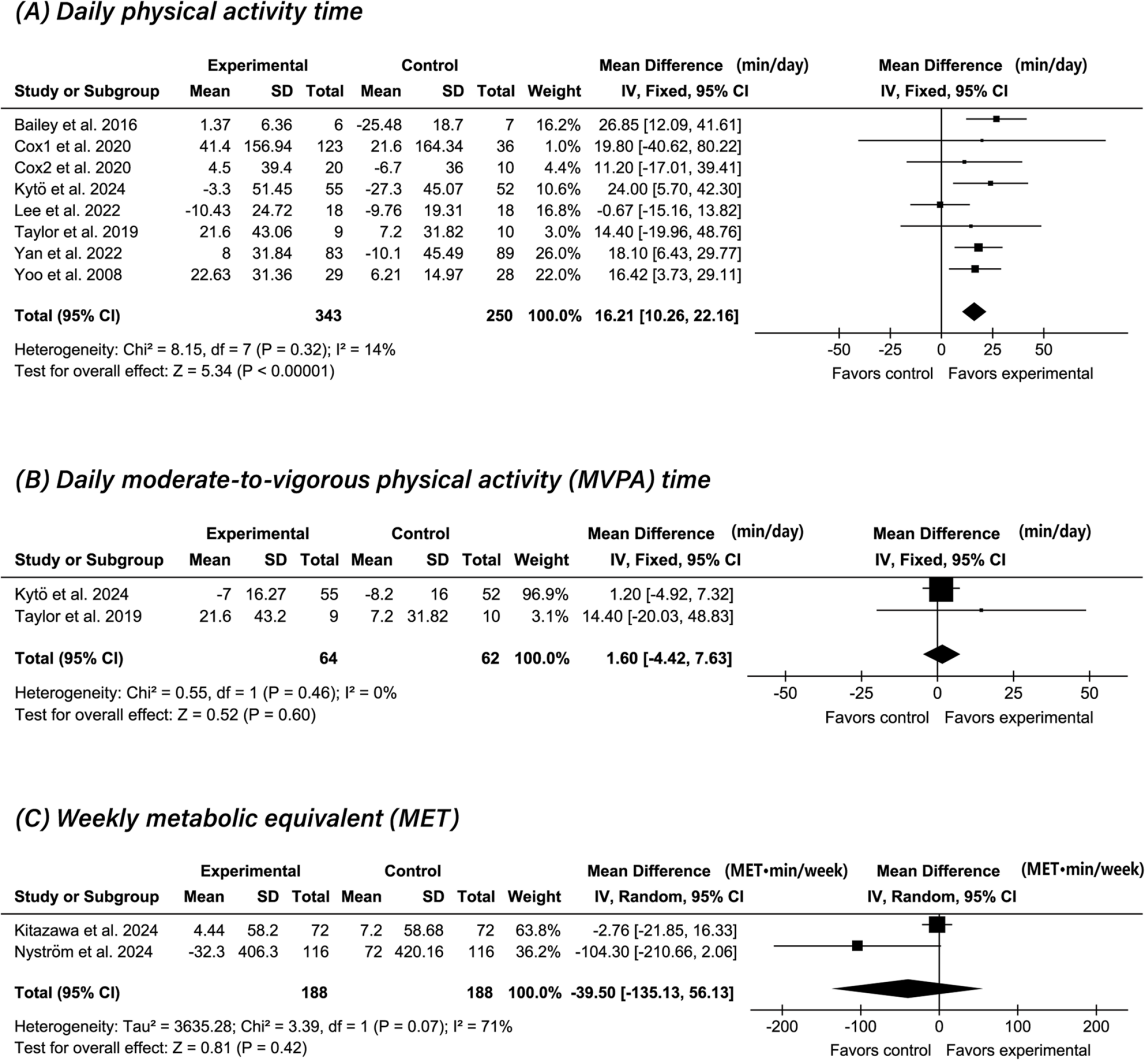
Comparison of CGM intervention with controls on physical activity:

Eight studies (*n* = 593) evaluated the effect of CGM on daily physical activity time [16, 17, 37, 40, 41, 48, 50, 52]. The pooled analysis revealed a significant association between CGM use and increased physical activity time compared with controls (MD: 16.21 min/day; 95% CI: 10.26 to 22.16; *P* < 0.0001; I² = 14%) (Fig. 2A).

Two studies (*n* = 126) examined the effect of CGM on daily MVPA time [37, 48], whereas 2 others (*n* = 376) evaluated weekly MET expenditure [33, 44]. Pooled analyses showed no significant between-group differences in MVPA time change (MD: 1.60 min/day; 95% CI: −4.42 to 7.63; *P* = 0.60; I² = 0%) (Fig. 2B) or MET changes (MD: −39.50; 95% CI: −135.13 to 56.13; *P* = 0.42; I² = 71%) (Fig. 2C).

### Effectiveness of CGM on diet (meta-analysis)

**Fig. 3:**
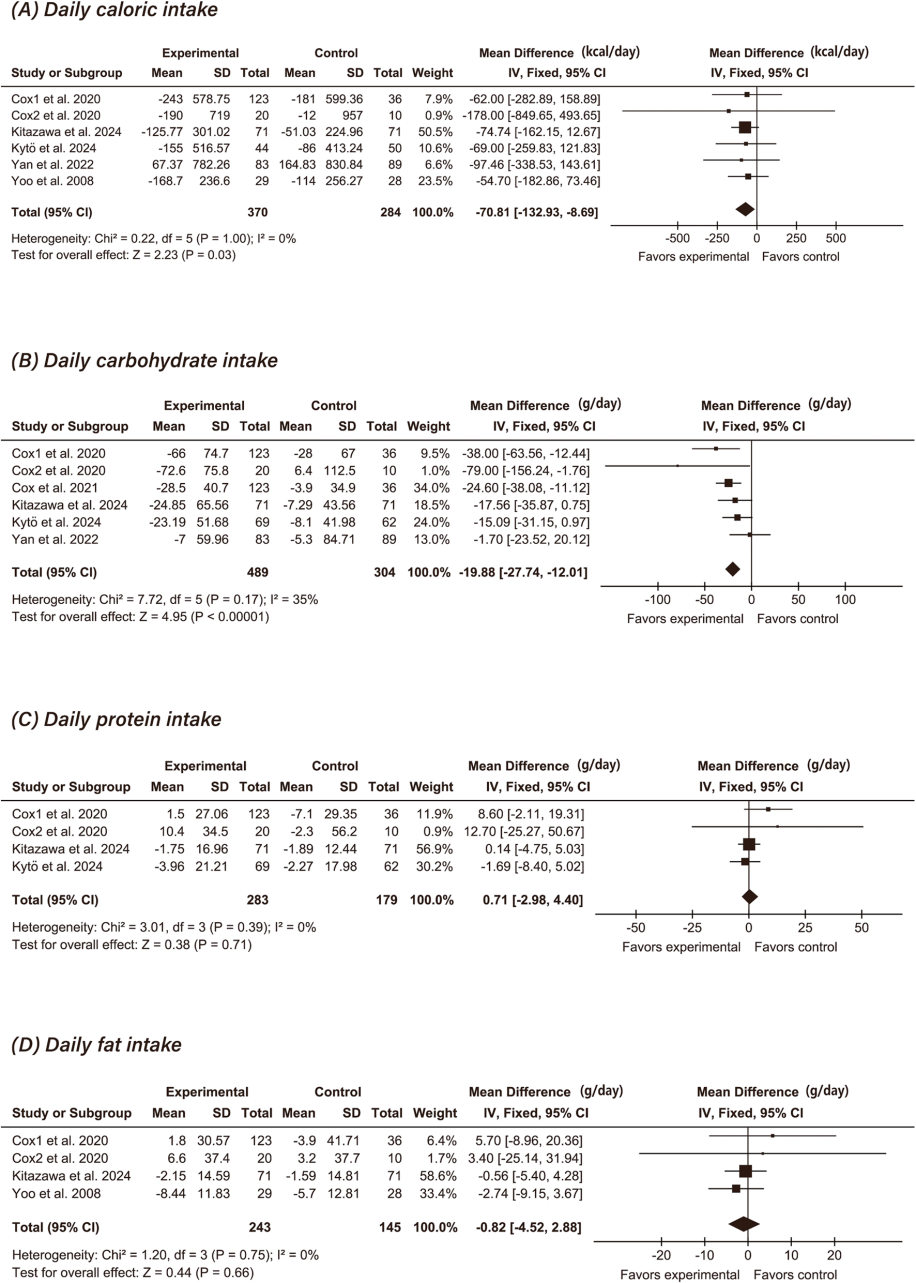
Comparison of CGM intervention with controls on dietary outcomes:

Six studies evaluated the effect of CGM on daily caloric (*n* = 654) [16, 17, 37, 41, 44, 50] and carbohydrate intake (*n* = 793) [17, 34, 37, 41, 44, 50]. The pooled analysis revealed a significant association between CGM use and reductions in both caloric intake (MD: −70.81 kcal/day; 95% CI: −132.93 to −8.69; *P* = 0.03; I² = 0%) (Fig. 3A) and carbohydrate consumption (MD: −19.88 g/day; 95% CI: −27.74 to −12.01; *P* < 0.00001; I² = 35%) (Fig. 3B) compared with controls.

Four studies (*n* = 462) assessed the impact of CGM on protein intake [17, 37, 41, 44] and fat intake (*n* = 388) [16, 17, 41, 44]. The pooled analysis showed no significant between-group differences in changes in daily protein consumption (MD: 0.71 g/day; 95% CI: −2.98 to 4.40; *P* = 0.71; I² = 0%) (Fig. 3C) or fat consumption (MD: −0.82 g/day; 95% CI: −4.52 to 2.88; *P* = 0.66; I² = 0%) (Fig. 3D).

Analysis of meal frequency was restricted due to limited data availability; only 2 studies (*n* = 208) reported daily meal counts [50, 52]. Between-group comparisons were not feasible due to insufficient study contributions (Supplementary Fig. 3A).

### Sensitivity analysis

Between-group comparisons showed low heterogeneity for most outcomes (I² = 14% for daily MVPA time; I² = 0% for daily caloric, protein, and fat intake). Carbohydrate intake had moderate heterogeneity (I² = 35%) and was evaluated with a leave-one-out sensitivity analysis, confirming a robust pooled effect (MD: −17.44 to −22.59 g/day, *P* ≤ 0.0004). Heterogeneity was mainly driven by Yan et al. 2022, whose exclusion reduced I² to 14%, while other studies had minimal impact. Weekly MET changes showed high heterogeneity (I² = 71%), but sensitivity analysis was not performed due to only two studies.

### Risk of bias

**Fig. 4:**
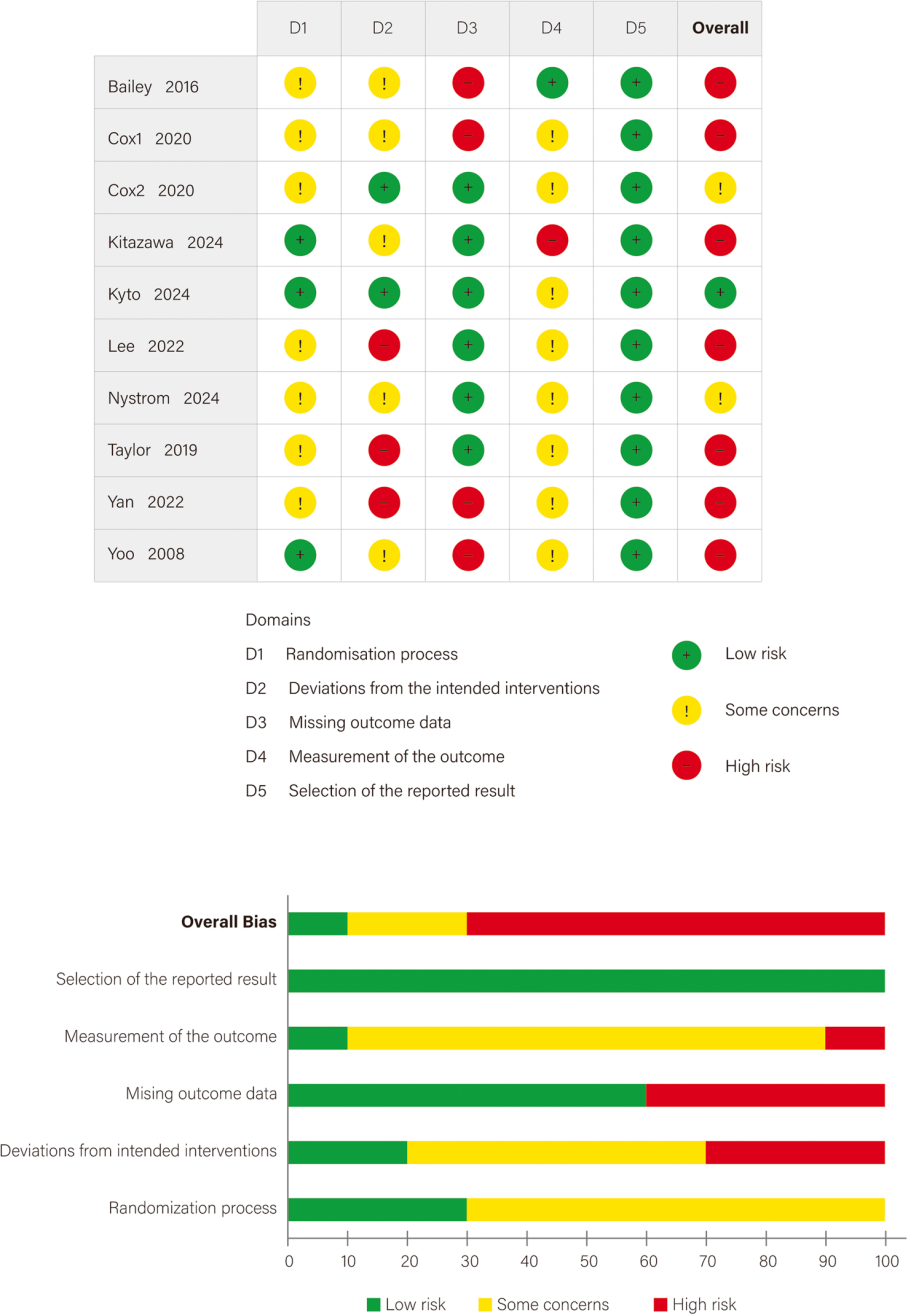
Risk of bias summary for physical activity outcomes

**Fig. 5:**
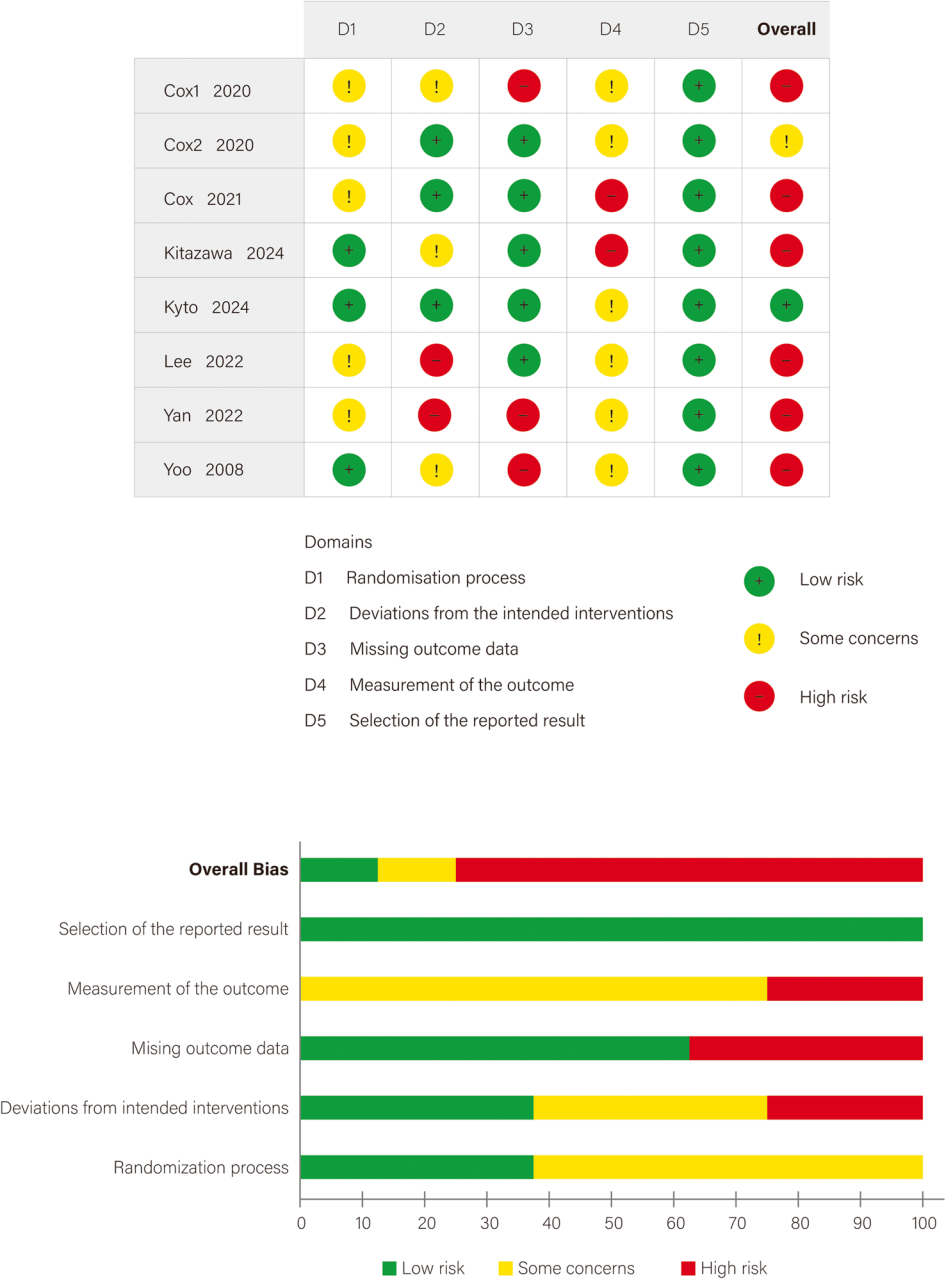
Risk of bias summary for dietary outcomes

The risk of bias results were presented in Figs. 4 and 5, with detailed assessments provided in Supplementary Tables 4 and 5. Although most studies claimed randomization, many did not describe the procedures, resulting in a rating of “some concerns.” Most interventions involved unblinded CGM combined with CGM-guided health education. Blinding was largely infeasible due to the nature of CGM and CGM-guided behavioral education, leading to high risk of bias in this domain. As observing glucose levels and adjusting behaviors accordingly are intended components of the intervention, these actions were not considered as sources of bias. Additionally, several studies did not report intention-to-treat analyses and appeared to rely on per-protocol approaches, further increasing the risk of bias. As a result, 6 of the 8 diet-related studies and 7 of the 10 physical activity–related studies were rated as high risk.

### Quality of evidence

We assessed the quality of evidence for both primary and secondary outcomes. Although the inconsistency and indirectness of the research are not serious, most of the results show issues with imprecision and a high risk of bias, leading to an overall low or very low quality of evidence. Detailed evaluations are provided in Table 2.

**Table 2:** Quality of evidence assessed using the GRADE approach

Outcomes	Certainty assessment						Effect	Certainty	Summary of findings
	Participants (No. of studies study design)	Risk of bias^a^	Inconsistency^b^	Indirectness	Imprecision^c^	Other consideration	MD (95% CI)		Importance
Physical activity									
Daily PA time (min/day)	593 (8RCT)	Very serious	Not serious	Not serious	Not serious	None	MD 16.21 (10.26 to 22.16)	⨁⨁◯◯ Low	Critical
Daily MVPA time (min/day)	126 (2RCT)	Serious	Not serious	Not serious	Very serious	None	MD 1.60 (−4.42 to 7.63)	⨁◯◯◯ Very low	Critical
Weekly METs (min/week)	376(2RCT)	Very serious	Serious	Not serious	Very serious	None	MD −39.50 (−135.13 to 56.13)	⨁◯◯◯ Very low	Critical
Diet									
Caloric intake (kcal/day)	654 (6RCT)	Very serious	Not serious	Not serious	Not serious	None	MD −70.81 (−132.93 to −8.69)	⨁⨁◯◯ Low	Critical
Carbohydrate intake (g/day)	793(6RCT)	Very serious	Not serious	Not serious	Not serious	None	MD −19.88 (−27.74 to −12.01)	⨁⨁◯◯ Low	Critical
Protein intake (g/day)	462(4RCT)	Serious	Not serious	Not serious	Serious	None	MD 0.71 (−2.98 to 4.40)	⨁⨁◯◯ Low	Important
Fat intake (g/day)	388(4RCT)	Very serious	Not serious	Not serious	Serious	None	MD −0.82 (−4.52 to 2.88)	⨁◯◯◯ Very low	Important
Number of meals (times/day)	208(2 RCT)	Very serious	Not serious	Not serious	Very serious	None	MD 0.05 (−0.13 to 0.23)	⨁◯◯◯ Very low	Important

*GRADE* Grading of Recommendations Assessment, Development and Evaluation, *MD* mean difference, *CI* confidence interval, *RCT* randomized controlled trial, *PA* physical activity, *MVPA* moderate to vigorous physical activity, *MET* metabolic equivalent. ^a^ When risk of bias was assessed with Cochrane RoB 2 tool. ^b^ The I^2^ was used to assess inconsistency across the results. ^c^ Confidence intervals were wide or sample size was < 400, possibly causing imprecise estimates

## Discussion

This study indicates that the use of CGM is associated with improvements in lifestyle behaviors among adults with diabetes, including increases in daily physical activity time and reductions in caloric and carbohydrate intake. Although these findings suggest that CGM may serve not only as a glycemic monitoring tool but also as a behavioral feedback mechanism that supports self-regulation and encourages healthier lifestyle choices, the certainty of evidence supporting these effects is low.

Of the 28 studies included in this systematic review, the majority demonstrated that CGM use was associated with favorable changes in health behaviors of people with diabetes. Evidence from non-randomized studies highlighted the breadth of this positive behavioral change across different populations. Findings from five cohort studies indicated that CGM use promoted healthier dietary and physical activity behaviors among individuals with T1D [38, 39, 49], T2D [49, 51], and prediabetes [51]; of note, two cohort studies specifically reported effects in children with T1D [38, 39], though the meta-analysis included only adult participants. Collectively, these non-RCT findings suggest that CGM may serve as a broadly applicable behavioral intervention tool for people with various types of diabetes. In contrast, most RCTs included in the meta-analysis focused on adults with T2D, with only two involving individuals with T1D and one including pregnant women with diabetes. Consequently, the quantitative results primarily reflect CGM’s effects in T2D adults. Additional RCTs are warranted to clarify its behavioral impact among those with T1D, including children and adolescents, as well as gestational or pregnancy-related diabetes.

In terms of physical activity, the CGM group demonstrated a significantly greater increase in daily physical activity time change compared with the control group, with a between-group difference in change of 16.21 min (low-certainty evidence). However, between-group differences in MVPA time change (increase of 1.6 min) (very low-certainty evidence) and MET value change (decrease of 39.5 units) (very low-certainty evidence) did not reach statistical significance, suggesting that the observed improvements were mainly due to increases in lower-intensity activity rather than structured vigorous exercise. However, the sustainability of these effects remains unclear, as the included trials for meta-analysis were short (7–13 weeks) and lacked long-term follow-up. Although one case–control study reported benefits over six months, it did not provide quantitative results [43]. Sedentary behaviors and physical inactivity are well-established risk factors for the onset and progression of diabetes [54]. Regular physical activity improves insulin sensitivity in people with diabetes, and this benefit may persist for more than 72 h after the last exercise session [55]. Even in sedentary individuals with insulin resistance, short periods of physical activity can enhance insulin sensitivity [56]. Despite these benefits, only 39% of individuals with diabetes engage in regular physical activity [57, 58]. Fear of hypoglycemia remains a major barrier to exercise adoption [59]. This fear often leads individuals with diabetes to choose low-intensity activities over clinically recommended MVPA regimens [60]. CGM’s real-time monitoring and alert functions may reduce the risk of exercise-induced hypoglycemia, helping alleviate fear and improve confidence in physical activity [42, 59]. Visual feedback showing post-exercise glucose improvements may further motivate engagement [32]. CGM data can also support personalized exercise prescriptions by enabling clinicians to tailor activity type and intensity based on glucose fluctuations. Clinically, integrating CGM feedback into exercise counseling may help individuals plan safe and effective activity while minimizing hypoglycemia risk. At the policy level, incorporating CGM-supported exercise management into diabetes care programs may enhance adherence and improve resource efficiency. For people with diabetes, routinely reviewing CGM patterns before and after exercise may reinforce the benefits of physical activity and support ongoing participation. Together, CGM may help break the cycle of fear-avoidance and poor glucose control, offering a promising tool to strengthen exercise interventions in diabetes management.

Regarding dietary outcomes, our quantitative analysis showed that CGM significantly influenced patients’ overall energy intake as well as their consumption of individual macronutrients. Compared with controls, the CGM group showed a significantly greater reduction in daily caloric intake change (− 70.81 kcal/day) (low-certainty evidence) and carbohydrate consumption change (− 19.88 g/day) (low-certainty evidence). CGM may promote dietary improvements through three mechanisms. First, CGM’s real-time visualization function enables individuals to observe the dynamic effects of different foods, particularly delayed peaks and prolonged elevations after high–glycemic index meals. Second, dynamic glucose patterns help individuals understand how meal timing and food sequence influence glycemic control, improving adherence to dietary guidance [15, 61]. Third, objective CGM data allow nutrition professionals to provide personalized nutrition plans that reduce carbohydrate and caloric intake without increasing medication reliance [15]. Evidence suggests particular benefit in gestational diabetes, where CGM-guided nutrition is linked to reduced macrosomia risk [8]. Importantly, one study reported sustained dietary improvements up to 13 months after discontinuation of CGM use [34, 41]. It is essential to emphasize that CGM-guided dietary management should focus on achieving good glycemic control while ensuring adequate nutrition, rather than imposing excessive carbohydrate or calorie restrictions that could increase the risk of hypoglycemia, especially in older adults. Clinically, integrating CGM data into routine nutrition counseling may help identify problematic foods and patterns. At a policy level, reimbursement for CGM-supported dietary management may enhance adherence and support long-term diabetes control. For individuals, reviewing post-meal glucose trends can reinforce healthy eating and strengthen self-management. Overall, CGM shows promise for translating real-time glucose data into meaningful dietary behavior change.

Diabetes requires lifelong self-management [62], yet adherence to recommended lifestyle behaviors remains suboptimal [63], particularly regarding physical activity and dietary improvements [64, 65]. In-depth analysis has shown that some people with diabetes using CGM improve their glycemic control primarily by enhancing medication adherence, rather than by making proactive behavior changes [31, 33, 48, 52]. This technical monitoring alone strategy may be insufficient for achieving sustainable diabetes control in the absence of behavior change. Effective CGM use relies on three components: [1] consistent use of reliable sensor technology [2], structured and personalized educational support, and [3] proficiency of people with diabetes in interpreting and applying data [47]. CGM-based education plays a central role by helping individuals translate real-time feedback into meaningful adjustments in diet and physical activity [14, 66]. Integrating such education into routine care may strengthen self-management skills and support long-term improvements in glycemic control.

The continued evolution of CGM technology is propelling diabetes care toward a new era of intelligence and precision. Future CGM systems are expected to integrate with multimodal health monitoring tools—including wearable devices and AI-driven applications—enabling synchronous tracking of glucose, physical activity, dietary intake, and sleep patterns, while supporting real-time prediction of adverse events such as hypoglycemia [20, 67]. These advancements will enable the synchronous monitoring of blood glucose, physical activity, dietary intake, and sleep patterns, while supporting risk prediction systems that can identify potential adverse events, such as hypoglycemia, in advance. Moreover, CGM will increasingly interface with online digital healthcare platforms to facilitate data sharing and multidisciplinary collaboration. Through remote coordination, endocrinologists, pharmacists, nutritionists, psychologists, and other specialists will be able to design and adjust personalized lifestyle intervention plans. This closed-loop “monitor-analysis-act” model has the potential to reduce healthcare burdens, enhance management efficiency, and ultimately support the creation of a patient-centered, intelligent diabetes management system [44].

### Limitations of the study

This study has several limitations. First, most of the included RCTs were not specifically designed to evaluate the effects of CGM on physical activity or dietary outcomes. Second, the limited number of available studies precluded subgroup analysis that could have clarified differences in behavioral impacts across CGM types or diabetes subtypes. For outcomes such as daily MET, the small number of studies and substantial heterogeneity further limit the reliability of the quantitative estimates. In addition, because the quantitative meta-analysis was primarily based on studies involving participants with T2D, the generalizability of these results to individuals with T1D or gestational diabetes may be limited. Moreover, the assessment methods for physical activity and dietary intake varied considerably across studies, and most relied on self-reported data. Although data were standardized into common units and indicators for analysis, systematic errors arising from measurement heterogeneity cannot be ruled out. Furthermore, the inherent characteristics of CGM interventions posed design challenges, such as the impracticality of double-blinding and allocation concealment. Along with other methodological limitations (e.g., small sample sizes and potential reporting bias), these factors contributed to the overall high risk of bias and the low to very low certainty of evidence. Finally, although most studies did not report conflicts of interest, 16 were funded by CGM manufacturers and 9 disclosed author conflicts. These potential sources of bias should be considered when interpreting the results.

### Strengths of the study

Despite these limitations, the study has several strengths. It is the first meta-analysis to quantitatively evaluate the impact of CGM on behavioral management in diabetes. The research team conducted detailed classification and processing of raw data, thereby enhancing the rigor and reliability of the analysis. Moreover, the certainty of evidence for each outcome was systematically assessed to produce evidence-based results.

## Conclusions

In conclusion, this systematic review and meta-analysis found that CGM use can facilitate positive changes in physical activity and dietary behaviors across different types of diabetes, as suggested by the qualitative evidence. Quantitative analysis indicated that CGM was associated with increased daily physical activity time and reduced daily caloric and carbohydrate intake, particularly among people with T2D. However, as the overall certainty of evidence was rated as low for physical activity changes, caloric and carbohydrate intake, these findings should be interpreted with caution. Nevertheless, this work provides actionable insights for clinicians, policymakers, and people with diabetes, potentially informing integrated diabetes care guidelines. Future high-quality, large-scale randomized controlled trials are warranted to confirm these associations and to clarify the mechanisms underlying CGM’s behavioral effects across diverse diabetes populations.

## Supplementary Information

Supplementary Material 1: Literature search strategy

Supplementary Material 2: PRISMA checklist

Supplementary Material 3: Supplementary Fig. 1. Differences in physical activity between baseline and post-intervention within the CGM group

Supplementary Material 4: Supplementary Fig. 2. Differences in dietary intake between baseline and post-intervention within the CGM group

Supplementary Material 5: Supplementary Fig. 3. Effects of CGM on the daily number of meals

Supplementary Material 6: Supplementary Table 1. Summary of excluded studies with reasons after full-text review

Supplementary Material 7: Supplementary Table 2. Review process for including RCTs in the meta-analysis

Supplementary Material 8: Supplementary Table 3. Sensitivity analysis using the leave-one-out method

Supplementary Material 9: Supplementary Table 4. Risk of bias (ROB 2) assessments for physical activity outcomes, with detailed justifications

Supplementary Material 10: Supplementary Table 5. Risk of bias (ROB 2) assessments for dietary outcomes, with detailed justifications

## Data Availability

The datasets used and/or analysed during the current study are available from the corresponding author on reasonable request.

## Abbreviations

CGM: Continuous glucose monitoring
T2D: type 2 diabetes
SMBG: self-monitoring of blood glucose
PRISMA: Preferred Reporting Items for Systematic Reviews and Meta-Analyses
MeSH: Medical Subject Headings
HbA1c: Hemoglobin A1c
NGSP: National Glycohemoglobin Standardization Program
MET: metabolic equivalent
MVPA: moderate to vigorous physical activity
RoB 2: Cochrane Risk of Bias 2 tool
GRADE: Grading of Recommendations Assessment, Development and Evaluation
GDT: Guideline Development Tool
RevMan: Review Manager
MD: mean difference
CI: confidence interval
RCT: randomized controlled trial
T1D: type 1 diabetes
IPAQ: International Physical Activity Questionnaire
SEBS: Self-efficacy for Exercise Behavior Scale
DSMQ: Diabetes Self-Management Questionnaire
CHMS: Canadian Health Measures Survey
7-day PAR: 7-day Physical Activity Recall Questionnaire
ASA24: Automated Self-Administered 24-hour Dietary Assessment Tool
EAT: Eating Assessment Test
DVS: Dietary Variety Score
