# Supplementary material for: Effects of continuous glucose monitoring on physical activity and diet in diabetes: a systematic review and meta-analysis
Source: Int J Behav Nutr Phys Act. 2026 Jan 21;23:14. doi: 10.1186/s12966-025-01870-0 (PMC12918550; doi:10.1186/s12966-025-01870-0)
Supplement: Supplementary file 1 — Supplementary Material 1: Literature search strategy [file 12966_2025_1870_MOESM1_ESM.docx]

**Effects of Continuous Glucose Monitoring on physical activity and diet in Diabetes: A Systematic Review and Meta-analysis**

**PubMed literature search strategy**

1 continuous glucose monitoring [MeSH Terms] (270)

2 continuous glucose monitor* (12992)

3 CGM (5610)

4 real time continuous glucose monitor* (1692)

5 rtCGM (272)

6 rt-CGM (152)

7 flash glucose monitoring (769)

8 FGM (2116)

9 intermittent* scanned continuous glucose monitor* (298)

10 isCGM (202)

11 is-CGM (13)

12 intermittent* viewed continuous glucose monitor* (17)

13 intermittent* continuous glucose monitor* (607)

14 iCGM (994)

15 sensor augmented pump (483)

16 SAP (18646)

17 1 OR 2 OR 3 OR 4 OR 5 OR 6 OR 7 OR 8 OR 9 OR 10 OR 11 OR 12 OR 13 OR 14 OR 15 OR 16 (36412)

18 exercise [MeSH Terms] (262369)

19 physical activ* (653862)

20 moderate-to-vigorous physical activity (9462)

21 MVPA (7288)

22 step* (987452)

23 daily step count* (2599)

24 steps per day (2955)

25 activity count* (386917)

26 energy expenditure (646775)

27 fitness (131388)

28 18 OR 19 OR 20 OR 21 OR 22 OR 23 OR 24 OR 25 OR 26 OR 27 (2731216)

29 diet [MeSH Terms] (344642)

30 diet* (1007405)

31 nutrition (860788)

32 low carbohydrate diet (12909)

33 29 OR 30 OR 31 OR 32 (1602263)

34 28 OR 34 (4058994)

35 diabetes mellitus [MeSH Terms] (535206)

36 diabetes (1011372)

37 mellitus (568141)

38 diabetes mellitus (639496)

39 34 OR 35 OR 36 OR 37 (1011431)

40 17 AND 34 AND 39 (2960)

**PubMed literature search strategy**

| **Database: PubMed-**Results (2960) | | |
| --- | --- | --- |
| Population terms | (((diabetes mellitus [MeSH Terms]) OR (diabetes)) OR (mellitus)) OR (diabetes mellitus) | 1011431 |
| Intervention terms | ((((((((((((((((continuous glucose monitoring [MeSH Terms]) OR (continuous glucose monitor*)) OR (CGM)) OR (real time continuous glucose monitor*)) OR (rtCGM)) OR (rt-CGM)) OR (flash glucose monitoring)) OR (FGM)) OR (intermittently scanned continuous glucose monitor*)) OR (intermittent* scanned continuous glucose monitor*))) OR (isCGM)) OR (is-CGM)) OR (intermittent* viewed continuous glucose monitor*)) OR (iCGM)) OR (sensor augmented pump)) OR (SAP) | 36412 |
| Outcome terms | ((((((((((exercise [MeSH Terms])) OR (physical activ*)) OR (moderate-to-vigorous physical activity)) OR (MVPA)) OR (step*)) OR (daily step count*)) OR (steps per day)) OR (activity count*)) OR (energy expenditure)) OR (fitness) (2731216)  (((diet[MeSH Terms]) OR (diet*)) OR (nutrition)) OR (low carbohydrate diet) (1602263) | 4058994 |

**Specific version of PubMed literature search strategy**

((((diabetes mellitus [MeSH Terms]) OR (diabetes)) OR (mellitus)) OR (diabetes mellitus)) AND ((((((diet[MeSH Terms]) OR (diet*)) OR (nutrition)) OR (low carbohydrate diet)) OR (((((((((((exercise [MeSH Terms])) OR (physical activ*)) OR (moderate-to-vigorous physical activity)) OR (MVPA)) OR (step*)) OR (daily step count*)) OR (steps per day)) OR (activity count*)) OR (energy expenditure)) OR (fitness))) AND (((continuous glucose monitoring [MeSH Terms]) OR continuous glucose monitor* OR CGM OR real time continuous glucose monitor* OR rtCGM OR rt-CGM OR flash glucose monitoring OR FGM OR intermittent* scanned continuous glucose monitor* OR isCGM OR is-CGM OR intermittent* viewed continuous glucose monitor* OR intermittent continuous glucose monitor* OR iCGM OR sensor augmented pump) OR SAP)) (2960)

("diabetes mellitus"[MeSH Terms] OR ("diabete"[All Fields] OR "diabetes mellitus"[MeSH Terms] OR ("diabetes"[All Fields] AND "mellitus"[All Fields]) OR "diabetes mellitus"[All Fields] OR "diabetes"[All Fields] OR "diabetes insipidus"[MeSH Terms] OR ("diabetes"[All Fields] AND "insipidus"[All Fields]) OR "diabetes insipidus"[All Fields] OR "diabetic"[All Fields] OR "diabetics"[All Fields] OR "diabets"[All Fields]) OR "mellitus"[All Fields] OR ("diabetes mellitus"[MeSH Terms] OR ("diabetes"[All Fields] AND "mellitus"[All Fields]) OR "diabetes mellitus"[All Fields])) AND (("diet"[MeSH Terms] OR "diet*"[All Fields] OR ("nutrition s"[All Fields] OR "nutritional status"[MeSH Terms] OR ("nutritional"[All Fields] AND "status"[All Fields]) OR "nutritional status"[All Fields] OR "nutrition"[All Fields] OR "nutritional sciences"[MeSH Terms] OR ("nutritional"[All Fields] AND "sciences"[All Fields]) OR "nutritional sciences"[All Fields] OR "nutritional"[All Fields] OR "nutritionals"[All Fields] OR "nutritions"[All Fields] OR "nutritive"[All Fields]) OR ("diet, carbohydrate restricted"[MeSH Terms] OR ("diet"[All Fields] AND "carbohydrate restricted"[All Fields]) OR "carbohydrate-restricted diet"[All Fields] OR ("low"[All Fields] AND "carbohydrate"[All Fields] AND "diet"[All Fields]) OR "low carbohydrate diet"[All Fields]) OR ("exercise"[MeSH Terms] OR (("physical examination"[MeSH Terms] OR ("physical"[All Fields] AND "examination"[All Fields]) OR "physical examination"[All Fields] OR "physical"[All Fields] OR "physically"[All Fields] OR "physicals"[All Fields]) AND "activ*"[All Fields]) OR ("moderate-to-vigorous"[All Fields] AND ("exercise"[MeSH Terms] OR "exercise"[All Fields] OR ("physical"[All Fields] AND "activity"[All Fields]) OR "physical activity"[All Fields])) OR "MVPA"[All Fields] OR "step*"[All Fields] OR (("dailies"[All Fields] OR "daily"[All Fields]) AND "step"[All Fields] AND "count*"[All Fields]) OR ("steps"[All Fields] AND ("potchefstroom electron law j"[Journal] OR "pediatr endocrinol rev"[Journal] OR "per"[All Fields]) AND "day"[All Fields]) OR (("activable"[All Fields] OR "activate"[All Fields] OR "activated"[All Fields] OR "activates"[All Fields] OR "activating"[All Fields] OR "activation"[All Fields] OR "activations"[All Fields] OR "activator"[All Fields] OR "activator s"[All Fields] OR "activators"[All Fields] OR "active"[All Fields] OR "actived"[All Fields] OR "actively"[All Fields] OR "actives"[All Fields] OR "activities"[All Fields] OR "activity s"[All Fields] OR "activitys"[All Fields] OR "motor activity"[MeSH Terms] OR ("motor"[All Fields] AND "activity"[All Fields]) OR "motor activity"[All Fields] OR "activity"[All Fields]) AND "count*"[All Fields]) OR ("energy metabolism"[MeSH Terms] OR ("energy"[All Fields] AND "metabolism"[All Fields]) OR "energy metabolism"[All Fields] OR ("energy"[All Fields] AND "expenditure"[All Fields]) OR "energy expenditure"[All Fields]) OR ("fitness"[All Fields] OR "fitnesses"[All Fields]))) AND ("continuous glucose monitoring"[MeSH Terms] OR (("continual"[All Fields] OR "continually"[All Fields] OR "continuance"[All Fields] OR "continuation"[All Fields] OR "continuations"[All Fields] OR "continue"[All Fields] OR "continued"[All Fields] OR "continuer"[All Fields] OR "continuers"[All Fields] OR "continues"[All Fields] OR "continuing"[All Fields] OR "continuities"[All Fields] OR "continuity"[All Fields] OR "continuous"[All Fields] OR "continuously"[All Fields]) AND ("glucose"[MeSH Terms] OR "glucose"[All Fields] OR "glucoses"[All Fields] OR "glucose s"[All Fields]) AND "monitor*"[All Fields]) OR "CGM"[All Fields] OR ("real"[All Fields] AND ("time"[MeSH Terms] OR "time"[All Fields]) AND ("continual"[All Fields] OR "continually"[All Fields] OR "continuance"[All Fields] OR "continuation"[All Fields] OR "continuations"[All Fields] OR "continue"[All Fields] OR "continued"[All Fields] OR "continuer"[All Fields] OR "continuers"[All Fields] OR "continues"[All Fields] OR "continuing"[All Fields] OR "continuities"[All Fields] OR "continuity"[All Fields] OR "continuous"[All Fields] OR "continuously"[All Fields]) AND ("glucose"[MeSH Terms] OR "glucose"[All Fields] OR "glucoses"[All Fields] OR "glucose s"[All Fields]) AND "monitor*"[All Fields]) OR "rtCGM"[All Fields] OR "rt-CGM"[All Fields] OR (("flash"[All Fields] OR "flash s"[All Fields] OR "flashed"[All Fields] OR "flashes"[All Fields] OR "flashing"[All Fields] OR "flashings"[All Fields]) AND ("glucose"[MeSH Terms] OR "glucose"[All Fields] OR "glucoses"[All Fields] OR "glucose s"[All Fields]) AND ("monitor"[All Fields] OR "monitor s"[All Fields] OR "monitorable"[All Fields] OR "monitored"[All Fields] OR "monitoring"[All Fields] OR "monitoring s"[All Fields] OR "monitorings"[All Fields] OR "monitorization"[All Fields] OR "monitorize"[All Fields] OR "monitorized"[All Fields] OR "monitors"[All Fields])) OR "FGM"[All Fields] OR ("intermittent*"[All Fields] AND ("radionuclide imaging"[MeSH Terms] OR ("radionuclide"[All Fields] AND "imaging"[All Fields]) OR "radionuclide imaging"[All Fields] OR "scanning"[All Fields] OR "scan s"[All Fields] OR "scanned"[All Fields] OR "scannings"[All Fields] OR "scans"[All Fields]) AND ("continual"[All Fields] OR "continually"[All Fields] OR "continuance"[All Fields] OR "continuation"[All Fields] OR "continuations"[All Fields] OR "continue"[All Fields] OR "continued"[All Fields] OR "continuer"[All Fields] OR "continuers"[All Fields] OR "continues"[All Fields] OR "continuing"[All Fields] OR "continuities"[All Fields] OR "continuity"[All Fields] OR "continuous"[All Fields] OR "continuously"[All Fields]) AND ("glucose"[MeSH Terms] OR "glucose"[All Fields] OR "glucoses"[All Fields] OR "glucose s"[All Fields]) AND "monitor*"[All Fields]) OR "isCGM"[All Fields] OR "is-CGM"[All Fields] OR ("intermittent*"[All Fields] AND ("viewed"[All Fields] OR "viewing"[All Fields] OR "viewings"[All Fields] OR "views"[All Fields]) AND ("continual"[All Fields] OR "continually"[All Fields] OR "continuance"[All Fields] OR "continuation"[All Fields] OR "continuations"[All Fields] OR "continue"[All Fields] OR "continued"[All Fields] OR "continuer"[All Fields] OR "continuers"[All Fields] OR "continues"[All Fields] OR "continuing"[All Fields] OR "continuities"[All Fields] OR "continuity"[All Fields] OR "continuous"[All Fields] OR "continuously"[All Fields]) AND ("glucose"[MeSH Terms] OR "glucose"[All Fields] OR "glucoses"[All Fields] OR "glucose s"[All Fields]) AND "monitor*"[All Fields]) OR (("intermittant"[All Fields] OR "intermittence"[All Fields] OR "intermittencies"[All Fields] OR "intermittency"[All Fields] OR "intermittent"[All Fields] OR "intermittently"[All Fields]) AND ("continual"[All Fields] OR "continually"[All Fields] OR "continuance"[All Fields] OR "continuation"[All Fields] OR "continuations"[All Fields] OR "continue"[All Fields] OR "continued"[All Fields] OR "continuer"[All Fields] OR "continuers"[All Fields] OR "continues"[All Fields] OR "continuing"[All Fields] OR "continuities"[All Fields] OR "continuity"[All Fields] OR "continuous"[All Fields] OR "continuously"[All Fields]) AND ("glucose"[MeSH Terms] OR "glucose"[All Fields] OR "glucoses"[All Fields] OR "glucose s"[All Fields]) AND "monitor*"[All Fields]) OR "iCGM"[All Fields] OR (("sensor"[All Fields] OR "sensor s"[All Fields] OR "sensoric"[All Fields] OR "sensorics"[All Fields] OR "sensoring"[All Fields] OR "sensorization"[All Fields] OR "sensorized"[All Fields] OR "sensors"[All Fields]) AND ("augment"[All Fields] OR "augmentation"[All Fields] OR "augmentations"[All Fields] OR "augmented"[All Fields] OR "augmenting"[All Fields] OR "augments"[All Fields]) AND "pump"[All Fields]) OR "SAP"[All Fields]))

**Cochrane literature search strategy**

#1 MeSH descriptor: [Diabetes Mellitus] explode all trees 46752

#2 ‘diabetes’ OR ‘mellitus’ OR ‘diabetes mellitus’ 117038

#3 MeSH descriptor: [Continuous Glucose Monitoring] explode all trees 28

#4 ‘continuous glucose monitor*’ OR ‘CGM’ OR ‘real time continuous glucose monitor*’ OR ‘rtCGM’ OR ‘rt-CGM’ OR ‘flash glucose monitoring’ OR ‘FGM’ OR ‘intermittently scanned continuous glucose monitor*’ OR ‘intermittent* scanned continuous glucose monitor*’ OR ‘isCGM’ OR ‘is-CGM’ OR ‘intermittent* viewed continuous glucose monitor*’ OR ‘iCGM’ OR ‘sensor augmented pump’ OR ‘SAP’ 8375

#5 MeSH descriptor: [Exercise] explode all trees 39597

#6 ‘physical activ*’ OR ‘moderate-to-vigorous physical activity’ OR ‘MVPA’ OR ‘step*’ OR ‘daily step count*’ OR ‘steps per day’ OR ‘activity count*’ OR ‘energy expenditure’ OR ‘fitness’ 160135

#7 MeSH descriptor: [Diet] explode all trees 26996

#8 ‘diet*’ OR ‘nutrition’ OR ‘low carbohydrate diet’ 158431

#9 #1 OR #2 120096

#10 #3 OR #4 8375

#11 #5 OR #6 181090

#12 #7 OR #8 161624

#13 #11 OR #12 308334

#14 #9 AND #10 AND #13 1876

**Embase** **literature search strategy**

#14 #9 AND #10 AND #13 5483

#13 #11 OR #12 4403636

#12 #7 OR #8 2093501

#11 #5 OR #6 2595124

#10 #3 OR #4 58501

#9 #1 OR #2 1697320

#8 'diet*' OR 'nutrition' OR 'low carbohydrate diet' 2093408

#7 'diet'/exp 456953

#6 'physical activ*' OR 'moderate-to-vigorous physical activity' OR 'mvpa' OR 'step*' OR 'daily step count*' OR 'steps per day' OR 'activity count*' OR 'energy expenditure' OR 'fitness' 2256618

#5 'exercise'/exp 478739

#4 'continuous glucose monitor*' OR 'cgm' OR 'real time continuous glucose monitor*' OR 'rtcgm' OR 'rt-cgm' OR 'flash glucose monitoring' OR 'fgm' OR 'intermittently scanned continuous glucose monitor*' OR 'intermittent* scanned continuous glucose monitor*' OR 'iscgm' OR 'is-cgm' OR 'intermittent* viewed continuous glucose monitor*' OR 'icgm' OR 'sensor augmented pump' OR 'sap' 58501

#3 'continuous glucose monitoring'/exp 1484

#2 'diabetes' OR 'mellitus' OR 'diabetes mellitus'/exp OR 'diabetes mellitus' 1697320

#1 'diabetes mellitus'/exp 1372573

**Web of Science**

1 "TS=(diabetes OR mellitus OR diabetes mellitus OR diabetes mellitus) and Preprint Citation Index (Exclude – Database) "All Databases 1162008

2 "TS=(continuous glucose monitor* OR CGM OR real time continuous glucose monitor* OR rtCGM OR rt-CGM OR flash glucose monitoring OR FGM OR intermittently scanned continuous glucose monitor* OR intermittent* scanned continuous glucose monitor* OR isCGM OR is-CGM OR intermittent* viewed continuous glucose monitor* OR iCGM OR sensor augmented pump OR SAP ) and Preprint Citation Index (Exclude – Database) " All Databases 78140

3 "TS=(exercise OR physical activ* OR moderate-to-vigorous physical activity OR MVPA OR step* OR daily step count* OR steps per day OR activity count* OR energy expenditure OR fitness) and Preprint Citation Index (Exclude – Database)" All Databases 5425837

4 "TS=(diet OR diet* OR nutrition OR low carbohydrate diet) and Preprint Citation Index (Exclude – Database) "All Databases 2645934

5 "#4 OR #3 and Preprint Citation Index (Exclude – Database) " All Databases 7659878

6 "#5 AND #2 AND #1 and Preprint Citation Index (Exclude – Database)" All Databases 3123
