# Supplementary figures and images for: Effects of continuous glucose monitoring on physical activity and diet in diabetes: a systematic review and meta-analysis
Source: Int J Behav Nutr Phys Act. 2026 Jan 21;23:14. doi: 10.1186/s12966-025-01870-0 (PMC12918550; doi:10.1186/s12966-025-01870-0)

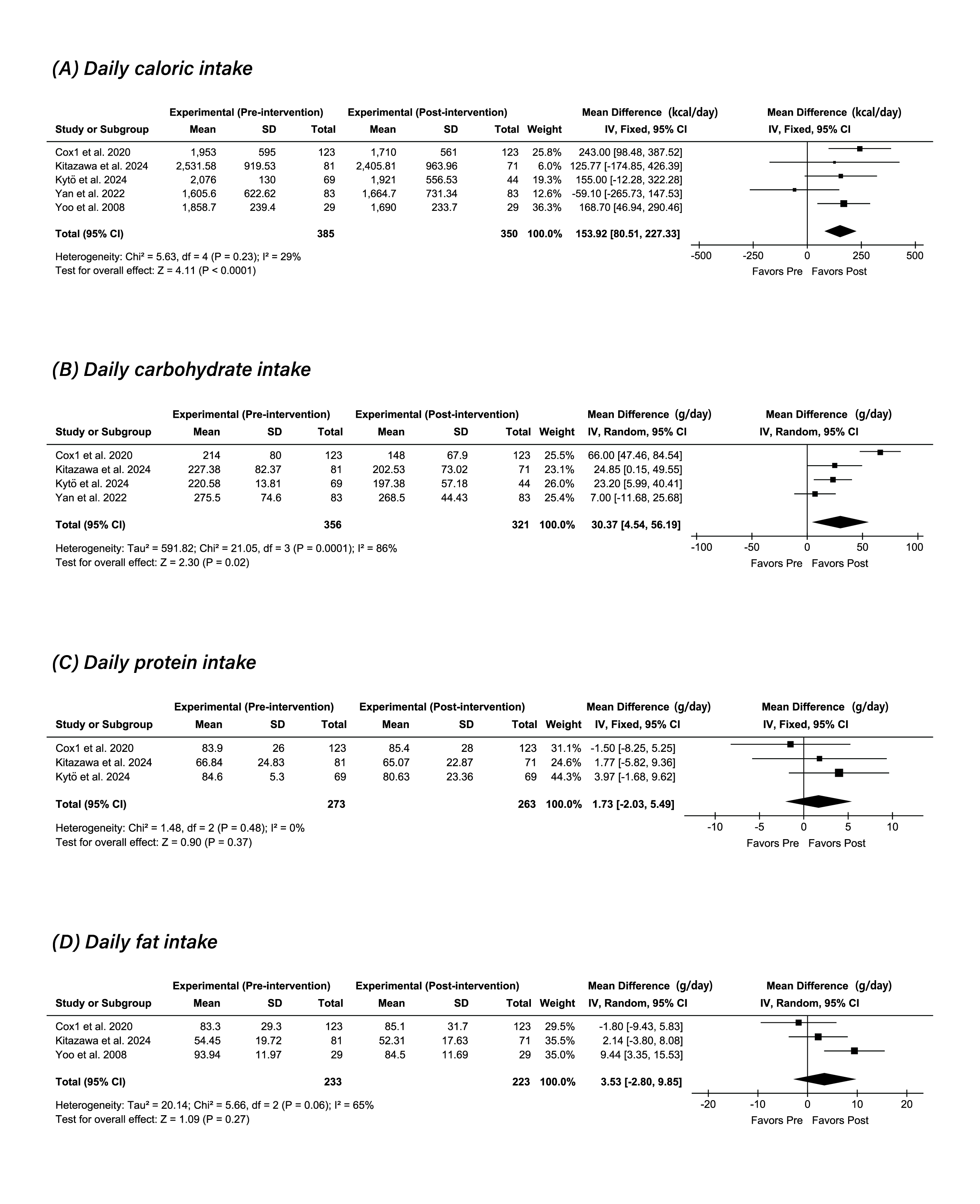

Supplement: Supplementary file 2 — Supplementary Material 2: PRISMA checklist [file 12966_2025_1870_MOESM2_ESM.tif]

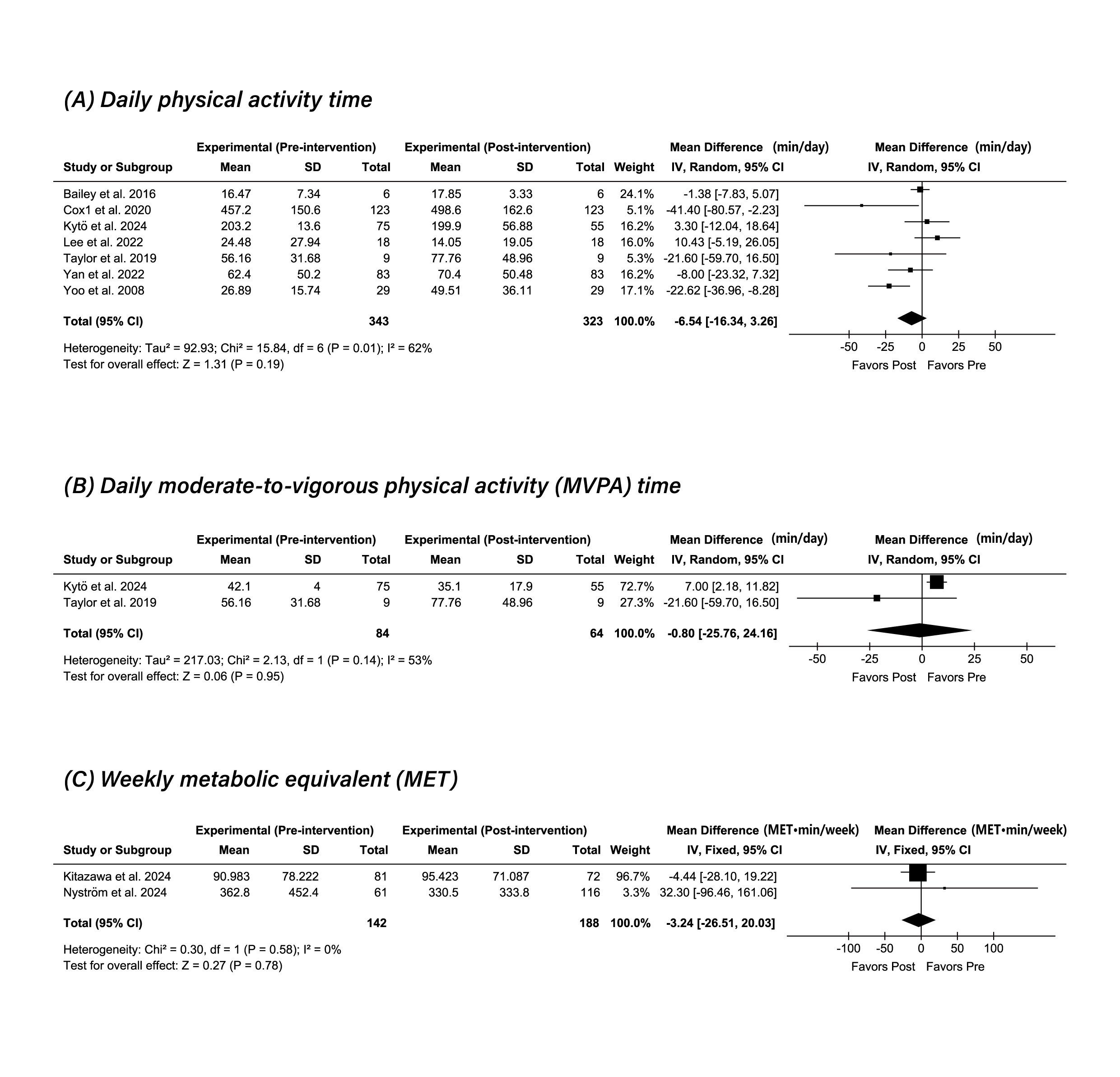

Supplement: Supplementary file 3 — Supplementary Material 3: Supplementary Fig. 1. Differences in physical activity between baseline and post-intervention within the CGM group [file 12966_2025_1870_MOESM3_ESM.tif]

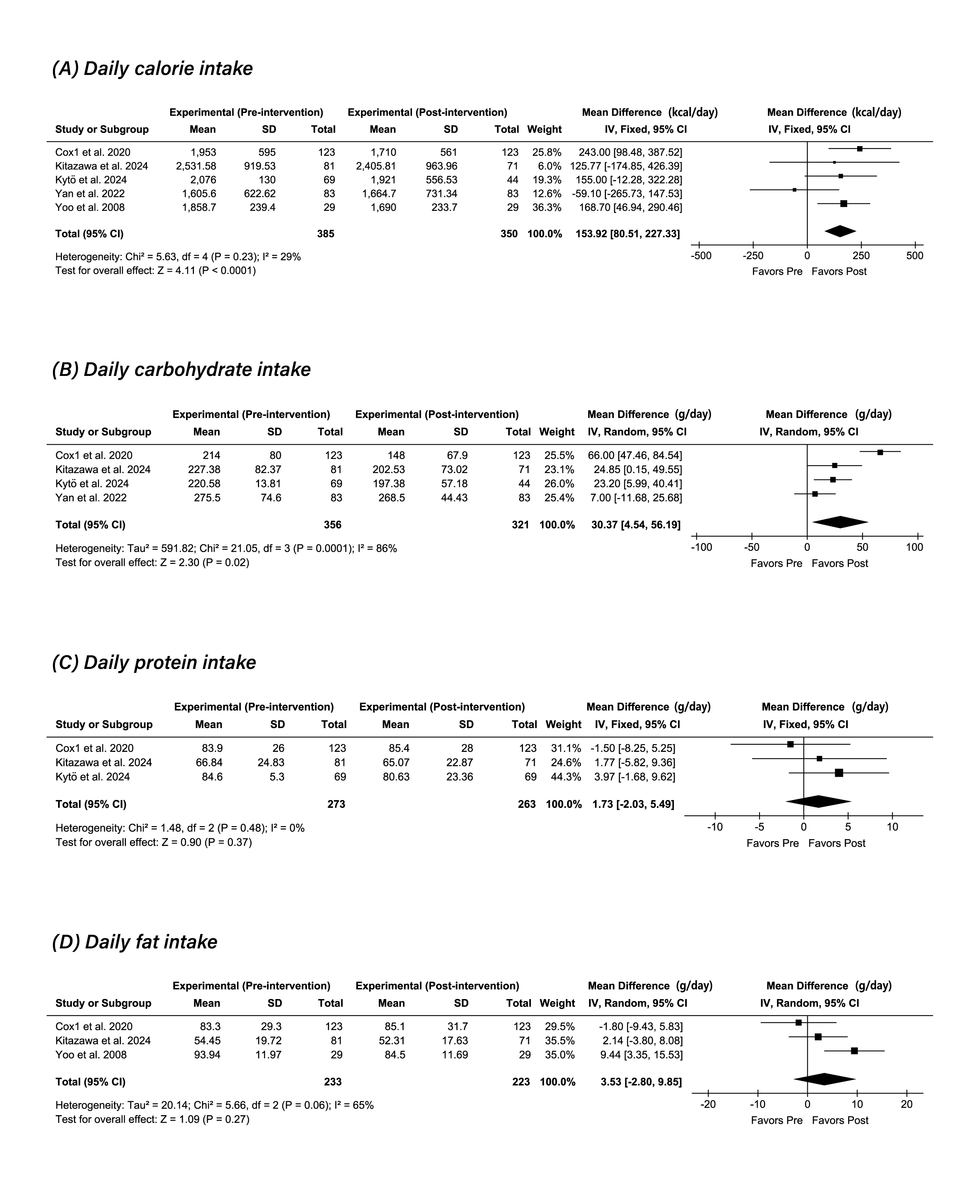

Supplement: Supplementary file 4 — Supplementary Material 4: Supplementary Fig. 2. Differences in dietary intake between baseline and post-intervention within the CGM group [file 12966_2025_1870_MOESM4_ESM.tif]

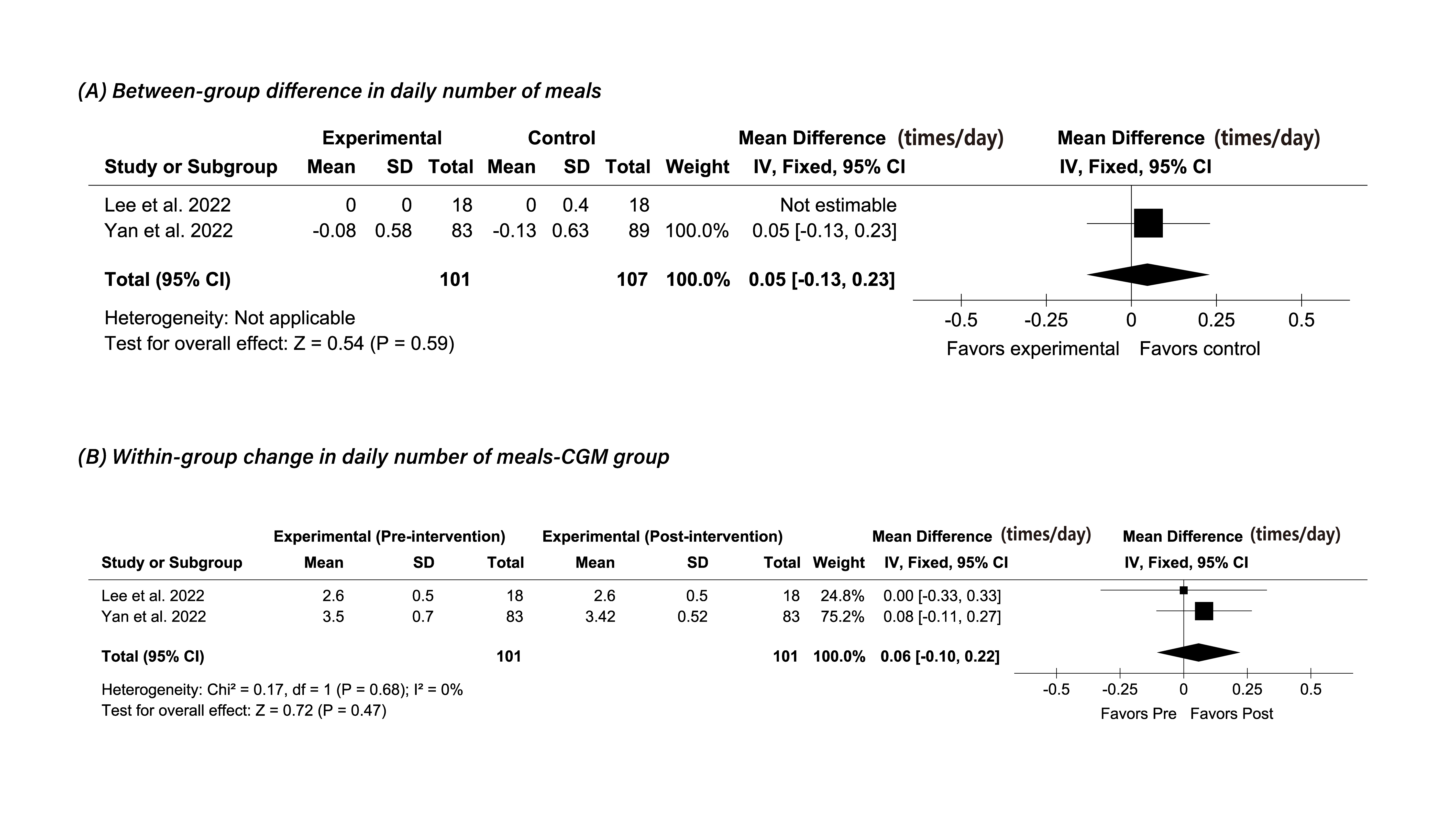

Supplement: Supplementary file 5 — Supplementary Material 5: Supplementary Fig. 3. Effects of CGM on the daily number of meals [file 12966_2025_1870_MOESM5_ESM.tif]
