# Supplementary material for: Effects of continuous glucose monitoring on physical activity and diet in diabetes: a systematic review and meta-analysis
Source: Int J Behav Nutr Phys Act. 2026 Jan 21;23:14. doi: 10.1186/s12966-025-01870-0 (PMC12918550; doi:10.1186/s12966-025-01870-0)
Supplement: Supplementary file 6 — Supplementary Material 6: Supplementary Table 1. Summary of excluded studies with reasons after full-text review [file 12966_2025_1870_MOESM6_ESM.docx]

Supplementary table 1 Summary of excluded studies with reason after reading whole papers.

| **Number** | **Authors** | **Year of publication** | **Country** | **Title** | **Journal** | **Volume** | **Issue** | **Pages** | **DOI** | **Exclusion reason** |
| --- | --- | --- | --- | --- | --- | --- | --- | --- | --- | --- |
| 1 | Anjum et al. | 2024 | Saudi Arabia | Optimizing type 2 diabetes management: AI-enhanced time series analysis of continuous glucose monitoring data for personalized dietary intervention | Peerj Computer Science | 10 | / | / | 10.7717/peerj-cs.1971 | No related outcomes |
| 2 | Benhamou et al. | 2023 | France | First Generation of a Modular Interoperable Closed-Loop System for Automated Insulin Delivery in Patients With Type 1 Diabetes: Lessons From Trials and Real-Life Data | Journal of Diabetes Science and Technology | 17 | 6 | 1433-1439 | 10.1177/19322968231186976 | No related outcomes |
| 3 | Carreiro et al. | 2016 | Brazil | Seventy two-hour glucose monitoring profiles in mild gestational diabetes mellitus: Differences from healthy pregnancies and influence of diet counseling | European Journal of Endocrinology | 175 | 3 | 201-209 | 10.1530/EJE-16-0015 | No related outcomes |
| 4 | Chen et al. | 2021 | China | Flash Glucose Monitoring Improves Glucose Control in People with Type 2 Diabetes Mellitus Receiving Anti-diabetic Drug Medication | Exp Clin Endocrinol Diabetes | 129 | 12 | 857-863 | 10.1055/a-0994-9850 | No related outcomes |
| 5 | Chesser et al. | 2024 | USA | Real-Time Continuous Glucose Monitoring in Adolescents and Young Adults With Type 2 Diabetes Can Improve Quality of Life | Journal of Diabetes Science and Technology | 18 | 4 | 911-919 | 10.1177/19322968221139873 | No related outcomes |
| 6 | Griauzde et al. | 2022 | USA | Continuous Glucose Monitoring With Low-Carbohydrate Nutritional Coaching to Improve Type 2 Diabetes Control: Randomized Quality Improvement Program | Journal of Medical Internet Research | 24 | 2 | / | 10.2196/31184 | No related outcomes |
| 7 | Jabbour et al. | 2024 | Qatar | Hypoglycemia avoidance behaviour in active Qatari adults with type 1 diabetes under blood glucose monitoring device | Diabetes Epidemiology and Management | 13 | / | / | 10.1016/j.deman.2023.100176 | Not suitable design |
| 8 | Jain et al. | 2021 | Canada | Glycemic improvement with a novel interim intervention technique using retrospective professional continuous glucose monitoring (GLITTER study): A study from Mumbai, India | Diabetes & Metabolic Syndrome-Clinical Research & Reviews | 15 | 3 | 703-709 | 10.1016/j.dsx.2021.03.011 | No related outcomes |
| 9 | Jospe et al. | 2020 | New Zealand | Teaching people to eat according to appetite - Does the method of glucose measurement matter? | Appetite | 151 | / | 104691 | 10.1016/j.appet.2020.104691 | Not suitable intervention |
| 10 | Kempf et al. | 2023 | Germany | Effectiveness of the Telemedical Lifestyle Intervention Program TeLIPro for Improvement of HbA1c in Type 2 Diabetes: a Randomized-Controlled Trial in a Real-Life Setting | Nutrients | 15 | 18 | / | 10.3390/nu15183954 | No related intervention |
| 11 | Lee et al. | 2023 | Korea | Clinical and Lifestyle Determinants of Continuous Glucose Monitoring Metrics in Insulin-Treated Patients with Type 2 Diabetes Mellitus | Diabetes & Metabolism Journal | 47 | 6 | 826-836 | 10.4093/dmj.2022.0273 | Not suitable design |
| 12 | Marlow et al. | 2023 | Australia | Healthy weight and overweight adolescents with type 1 diabetes mellitus do not meet recommendations for daily physical activity and sleep | Diabetes Research and Clinical Practice | 203 | / | / | 10.1016/j.diabres.2023.110879 | Not suitable design |
| 13 | Minuto et al. | 2021 | Italy | The Effect of Lockdown and Physical Activity on Glycemic Control in Italian Children and Young Patients With Type 1 Diabetes | Frontiers in Endocrinology | 12 | / | / | 10.3389/fendo.2021.690222 | Not suitable design |
| 14 | Molveau et al. | 2022 | Canada | Prevalence of nocturnal hypoglycemia in free-living conditions in adults with type 1 diabetes: What is the impact of daily physical activity? | Front Endocrinol | 13 | / | 953879 | 10.3389/fendo.2022.953879 | Not suitable design |
| 15 | Richardson et al. | 2024 | USA | Adding a Brief Continuous Glucose Monitoring Intervention to the National Diabetes Prevention Program: A Multimethod Feasibility Study | Journal of Diabetes Research | 2024 | / | / | 10.1155/2024/7687694 | Participants are prediabetes |
| 16 | Ruissen et al. | 2023 | the Netherlands | Clinical impact of an integrated e-health system for diabetes self-management support and shared decision making (POWER2DM): a randomised controlled trial | Diabetologia | 66 | 12 | 2213-2225 | 10.1007/s00125-023-06006-2 | No related outcomes |
| 17 | Schiel et al. | 2011 | Germany | An Innovative Telemedical Support System to Measure Physical Activity in Children and Adolescents with Type 1 Diabetes Mellitus | Experimental and Clinical Endocrinology & Diabetes | 119 | 9 | 565-568 | 10.1055/s-0031-1273747 | Not suitable design |
| 18 | Tornese et al. | 2020 | Italy | Glycemic Control in Type 1 Diabetes Mellitus During COVID-19 Quarantine and the Role of In-Home Physical Activity | Diabetes Technology and Therapeutics | 22 | 6 | 462-467 | 10.1089/dia.2020.0169 | Not suitable design |
| 19 | Whelan et al. | 2021 | UK | A digital lifestyle behaviour change intervention for the prevention of type 2 diabetes: a qualitative study exploring intuitive engagement with real-time glucose and physical activity feedback | Bmc Public Health | 21 | 1 | / | 10.1186/s12889-020-09740-z | Not suitable design |
| 20 | Yoo et al. | 2022 | Korea | Effect of structured individualized education on continuous glucose monitoring use in poorly controlled patients with type 1 diabetes: A randomized controlled trial | Diabetes Res Clin Pract | 184 | / | 109209 | 10.1016/j.diabres.2022.109209 | No related outcomes |
| 21 | Yost et al. | 2020 | USA | Continuous Glucose Monitoring With Low-Carbohydrate Diet Coaching in Adults With Prediabetes: Mixed Methods Pilot Study | JMIR Diabetes | 5 | 4 | e21551 | 10.2196/21551 | Participants are prediabetes |
