# Supplementary material for: Effects of continuous glucose monitoring on physical activity and diet in diabetes: a systematic review and meta-analysis
Source: Int J Behav Nutr Phys Act. 2026 Jan 21;23:14. doi: 10.1186/s12966-025-01870-0 (PMC12918550; doi:10.1186/s12966-025-01870-0)
Supplement: Supplementary file 7 — Supplementary Material 7: Supplementary Table 2. Review process for including RCTs in the meta-analysis [file 12966_2025_1870_MOESM7_ESM.docx]

**Supplementary Table 2. Review process for including RCTs in the meta-analysis.**

| **Number** | **Author** | **Title** | **Population** | **Intervention components** | **Control group** | **Physical activity measurements** | **Diet measurements** | **Meta-analysis for diet** | **Meta-analysis for PA** | **Highlight the effect of CGM** | **Not suitable for meta-analysis** |
| --- | --- | --- | --- | --- | --- | --- | --- | --- | --- | --- | --- |
| 1 | Ahn et al. | Effectiveness of Non-Contact Dietary Coaching in Adults with Diabetes or Prediabetes Using a Continuous Glucose Monitoring Device: A Randomized Controlled Trial | PreD, T2D | Unblinded CGM with nurse-led dietary education, feedback, individual and group coaching | Unblinded CGM with standard care (education on PA and medication adherence) | Not applicable | Weight Efficacy Lifestyle Questionnaire – Short Form |  |  | N | No CGM-focused intervention reported |
| 2^#^ | Allen et al. | Continuous glucose monitoring counseling improves physical activity behaviors of individuals with type 2 diabetes: A randomized clinical trial | T2D | Unblinded CGM (3 days, week 1) with retrospective CGM-based activity guidance and individualized education | Individualized diabetes education (90 minutes) | SEBS; accelerometers measuring frequency and intensity | Not applicable | No data for diet | Data cannot be merged |  | No suitable data |
| 3 | Allen et al. | A Continuous Glucose Monitoring and Problem-Solving Intervention to Change Physical Activity Behavior in Women with Type 2 Diabetes: A Pilot Study | T2D | CGM-based counseling on PA (week 1: glucose response and PA benefits); problem-solving counseling (week 4: addressing PA barriers) | CGM-based counseling on PA (week 1); attention-control diabetes education (week 4, without PA/diet components) | SEBS,  accelerometers (a product of movement frequency and intensity) | Subscale of the Summary of Diabetes Self-Care Activities |  |  | N | No CGM-focused intervention reported |
| 4 | Bailey et al. | Self-Monitoring Using Continuous Glucose Monitors with Real-Time Feedback Improves Exercise Adherence in Individuals with Impaired Blood Glucose: A Pilot Study | PreD, T2D | Unblinded CGM with instruction on self-monitoring of exercise and blood glucose, goal-setting, and using CGM to observe exercise effects on BG; exercise sessions twice weekly for 8 weeks | Standard care and exercise sessions twice weekly for 8 weeks | 7-day PARQ | Not applicable | No data for diet | √ | √ |  |
| 5 | Borel et al. | Closed-Loop Insulin Therapy for People With Type 2 Diabetes Treated With an Insulin Pump: A 12-Week Multicenter, Open-Label Randomized, Controlled, Crossover Trial | T2D | Hybrid closed-loop system: Accu-Chek Insight insulin pump with Dexcom G6 CGM | CSII (standard insulin pump therapy) and CGM | Mean daily PA (METs) from 1-week actimetry | Not applicable |  |  | N | No CGM-focused intervention reported |
| 6 | Cox1 et al. | Glycemic excursion minimization in the management of type 2 diabetes: a novel intervention tested in a randomized clinical trial | T2D | Unblinded CGM with prospective guidance on diet and PA, 6 hours of group training, and tracking of BG and PA. | Four 90-minute group sessions focused on reducing glucose excursions by education on diet and PA. | Blinded activity monitor (steps/day, hours active) | ASA24 | √ | √ | √ |  |
| 7 | Cox2 et al. | Minimizing Glucose Excursions (GEM) With Continuous Glucose Monitoring in Type 2 Diabetes: A Randomized Clinical Trial | T2D | Unblinded CGM with four group sessions on minimizing glycemic excursions, including a manual and diary addressing diet, activity, and hypoglycemia management | Routine care | Blinded activity monitor (Fitbit Charge 2); average daily active minutes | ASA24 | √ | √ | √ |  |
| 8 | Cox et al. | Long-term follow-up of a randomized clinical trial comparing glycemic excursion minimization (GEM) to weight loss (WL) in the management of type 2 diabetes | T2D | Unblinded CGM with four 90-minute group sessions over 3 months focused on reducing post-nutrient glucose excursions through dietary and PA guidance; no maintenance provided between post-assessment and follow-up | Four 90-minute group sessions focused on reducing post-nutrient glucose excursions through diet and PA education | Not applicable | Carbohydrates Routinely Consumed (servings) | √ | No data for PA | √ |  |
| 9 | Kitazawa et al. | Lifestyle Intervention With Smartphone App and isCGM for People at High Risk of Type 2 Diabetes: Randomized Trial | T2D | Unblinded CGM with Health 2Sync mobile app (tracking food and PA records, diabetes-related data; providing dietary and activity guidance based on isCGM data) | No lifestyle modification information; no use of healthcare-related smartphone applications | Japanese version of the IPAQ (short form) | Brief Diet History Questionnaire | √ | √ | √ |  |
| 10 | Kytö et al. | Periodic mobile application (eMOM) with self-tracking of glucose and lifestyle improves treatment of diet-controlled gestational diabetes without human guidance: a randomized controlled trial | GDM (24 to 28 weeks’ gestation) | Unblinded CGM combined with standard care and periodic use of a mobile application (eMOM) with wearable sensors, activity tracker, and food diary (1 week/month until delivery) | Standard care | Blinded accelerometer | Semiquantitative 142-item food-frequency questionnaire | √ | √ | √ |  |
| 11 | Lee et al. | FGM-based remote intervention for adults with type 1 diabetes: The FRIEND randomized clinical trial | T1D | Unblinded CGM with retrospective, individualized guidance on diet, medication, and PA | Unblinded CGM without intervention | Number of exercise sessions per week; hours of exercise per week | Number of meals and snacks per day | √ | √ | √ |  |
| 12^#^ | Majewska et al. | Flash glucose monitoring in gestational diabetes mellitus (FLAMINGO): a randomised controlled trial | GDM | FGM system with guidance on glycemic control, diet, and physical activity | SMBG with guidance on glycemic control, diet, and physical activity | Daily step count | Eating Assessment Test (EAT) | Data cannot be merged | Not report the data of PA |  | No suitable data. |
| 13 | Nyström et al. | Evaluation of Effects of Continuous Glucose Monitoring on Physical Activity Habits and Blood Lipid Levels in Persons With Type 1 Diabetes Managed With Multiple Daily Insulin Injections: An Analysis Based on the GOLD Randomized Trial (GOLD 8) | T1D with MDI | Unblinded CGM with guidance on insulin dosing, bolus correction, food choices, and effects of PA on glycemic control | SMBG with the same guidance provided | IPAQ | Not applicable | No data for diet | √ | √ |  |
| 14 | Taylor et al. | Efficacy of Real-Time Continuous Glucose Monitoring to Improve Effects of a Prescriptive Lifestyle Intervention in Type 2 Diabetes: A Pilot Study | T2D | Unblinded CGM with prospective guidance on diet and activity, tracking of glucose, diet, and PA, and lifestyle prescriptions | Blinded CGM without feedback and standard tracking and prescriptions | Seven-day ambulatory accelerometer: time spent in sedentary vs. moderate/vigorous activity | Not applicable | No data for diet | √ | √ |  |
| 15 | Yan et al. | Real-Time Flash Glucose Monitoring Had Better Effects on Daily Glycemic Control Compared With Retrospective Flash Glucose Monitoring in Patients With Type 2 Diabetes on Premix Insulin Therapy | T2D | Unblinded CGM with guidance on medication adjustment, tracking of diet and physical activity, individual education, and study logs (recording diet and exercise daily) | Blinded CGM (with feedback post-wear), diet and PA tracking, individual education, and study logs | Daily exercise time (min/day) | Energy intake, number of meals | √ | √ | √ |  |
| 16 | Yoo et al. | Use of a real time continuous glucose monitoring system as a motivational device for poorly controlled type 2 diabetes | T2D | Unblinded CGM with CGM-based guidance on diet and physical activity and individual education | Individual education and glucometer-based advice | Exercise time per week (min/week) | 3-day food records analyzed using Can-Pro 3.0 | √ | √ | √ |  |

PA, physical activity; PreD, prediabetes; T2D, type 2 diabetes; CGM, continuous glucose monitoring; SEBS, self-efficacy for exercise behavior; 7-day PARQ, 7-day physical activity recall questionnaire; CSII, continuous subcutaneous insulin infusion; MET, metabolic equivalent; ASA24, automated self-administered 24-hour recall; IPAQ, international physical activity questionnaire; GDM, gestational diabetes mellitus; T1D, type 1 diabetes; FGM, flash glucose monitoring; MDI, multiple daily insulin injections; SMBG, self-monitoring of blood glucose.
Notes: ^#^Two studies collected physical activity/dietary outcomes but did not provide extractable data for meta-analysis. Attempts to contact the study authors for additional information were unsuccessful.
