# Supplementary material for: Effects of continuous glucose monitoring on physical activity and diet in diabetes: a systematic review and meta-analysis
Source: Int J Behav Nutr Phys Act. 2026 Jan 21;23:14. doi: 10.1186/s12966-025-01870-0 (PMC12918550; doi:10.1186/s12966-025-01870-0)
Supplement: Supplementary file 8 — Supplementary Material 8: Supplementary Table 3. Sensitivity analysis using the leave-one-out method [file 12966_2025_1870_MOESM8_ESM.docx]

Supplementary table 3 Sensitivity analysis by the leave-one-out method

| **Study omitted** | **MD** | **LL** | **UL** | **I^2^** | **P** |
| --- | --- | --- | --- | --- | --- |
| **Diet Ex vs Co** |  |  |  |  |  |
| **Carbohydrates (g/day)** |  |  |  |  |  |
| Cox et al.2021 | -17.44 | -27.12 | -7.75 | 43% | 0.0004 |
| Cox1 et al.2020 | -17.98 | -26.25 | -9.71 | 28% | ＜0.00001 |
| Cox2 et al.2020 | -19.26 | -27.16 | -11.35 | 27% | ＜0.00001 |
| Kitazawa et al.2024 | -20.40 | -29.11 | -11.69 | 48% | ＜0.00001 |
| Kytö et al.2024 | -21.39 | -30.41 | -12.37 | 45% | ＜0.00001 |
| Yan et al.2022 | -22.59 | -31.02 | -14.16 | 14% | ＜0.00001 |
| **Diet Exp: Pre vs Post** |  |  |  |  |  |
| **Calories (kcal/day)** |  |  |  |  |  |
| Cox1 et al.2020 | 122.94 | 37.71 | 208.17 | 18% | 0.005 |
| Kitazawa et al.2024 | 155.7 | 80 | 231.41 | 46% | ＜0.0001 |
| Kytö et al.2024 | 153.66 | 71.96 | 235.36 | 47% | 0.0002 |
| Yan et al.2022 | 184.69 | 106.16 | 263.23 | 0% | ＜0.00001 |
| Yoo et al.2008 | 145.48 | 53.46 | 237.5 | 46% | 0.002 |
| **Carbohydrates (g/day)** |  |  |  |  |  |
| Cox1 et al.2020 | 17.65 | 6.39 | 28.92 | 0% | 0.002 |
| Kitazawa et al.2024 | 32.04 | -1.72 | 65.8 | 90% | 0.06 |
| Kytö et al.2024 | 32.79 | -4.53 | 70.11 | 90% | 0.09 |
| Yan et al.2022 | 38.36 | 9.46 | 67.25 | 84% | 0.009 |
| **Fat (g/day)** |  |  |  |  |  |
| Cox1 et al.2020 | 5.76 | -1.4 | 12.91 | 65% | 0.11 |
| Kitazawa et al.2024 | 4.06 | -6.94 | 15.07 | 80% | 0.47 |
| Yoo et al.2008 | 0.65 | -4.03 | 5.34 | 0% | 0.78 |
| **PA Exp: Pre vs Post** |  |  |  |  |  |
| **Daily PA time (min/day)** |  |  |  |  |  |
| Bailey et al.2016 | -9.01 | -22.55 | 4.53 | 66% | 0.19 |
| Cox1 et al.2020 | -4.54 | -13.76 | 4.68 | 59% | 0.33 |
| Kytö et al.2024 | -8.83 | -20.38 | 2.71 | 67% | 0.13 |
| Lee et al.2022 | -9.63 | -20.04 | 0.78 | 59% | 0.07 |
| Taylor et al.2019 | -5.75 | -15.98 | 4.49 | 67% | 0.27 |
| Yan et al.2022 | -6.73 | -18.51 | 5.05 | 68% | 0.26 |
| Yoo et al.2008 | -2.23 | -10.74 | 6.27 | 40% | 0.61 |

MD, mean difference; LL, lower limit; UL, upper limit; Ex, Experimental group; Co, Control group; Pre, Pre-intervention; Post, Post-intervention; PA, physical activity.
