# Supplementary material for: Effects of continuous glucose monitoring on physical activity and diet in diabetes: a systematic review and meta-analysis
Source: Int J Behav Nutr Phys Act. 2026 Jan 21;23:14. doi: 10.1186/s12966-025-01870-0 (PMC12918550; doi:10.1186/s12966-025-01870-0)
Supplement: Supplementary file 10 — Supplementary Material 10: Supplementary Table 5. Risk of bias (ROB 2) assessments for dietary outcomes, with detailed justifications [file 12966_2025_1870_MOESM10_ESM.docx]

Risk of bias for diet.

| **Unique ID** | Cox1 2020 | **Study ID** | DOI: 10.1136/bmjdrc-2020-001795 | **Assessor** |  |
| --- | --- | --- | --- | --- | --- |
| **Ref or Label** | BMJ Open Diabetes Res Care 2020 Dec;8(2):e001795. | **Aim** | assignment to intervention (the 'intention-to-treat' effect) |  |  |
| **Experimental** | CGM and CGM-based guidance on diet and activity | **Comparator** | Group education | **Source** | Journal article(s) |
| **Outcome** | A blinded activity monitor (steps/day, Hours active) | **Results** |  | **Weight** | 1 |
| **Domain** | **Signalling question** | | | **Response** | **Comments** |
| **Bias arising from the randomization process** | 1.1 Was the allocation sequence random? | | | NI |  |
|  | 1.2 Was the allocation sequence concealed until participants were enrolled and assigned to interventions? | | | NI |  |
|  | 1.3 Did baseline differences between intervention groups suggest a problem with the randomization process? | | | N | Participants in the different interventions did not differ on any demographic variables (table 1). |
|  | **Risk of bias judgement** | | | **Some concerns** |  |
| **Bias due to deviations from intended interventions** | 2.1.Were participants aware of their assigned intervention during the trial? | | | PY | Participants underwent different procedures that cannot be blinded. |
|  | 2.2.Were carers and people delivering the interventions aware of participants' assigned intervention during the trial? | | | PY |  |
|  | 2.3. If Y/PY/NI to 2.1 or 2.2: Were there deviations from the intended intervention that arose because of the experimental context? | | | NI |  |
|  | 2.4 If Y/PY to 2.3: Were these deviations likely to have affected the outcome? | | | NA |  |
|  | 2.5. If Y/PY/NI to 2.4: Were these deviations from intended intervention balanced between groups? | | | NA |  |
|  | 2.6 Was an appropriate analysis used to estimate the effect of assignment to intervention? | | | Y | Intent-to-treat analyses were performed using SAS V.9.4. A one-way analysis of variance compared changes in HbA1c among the four interventions. Orthogonal contrasts were used to divide the treatment sum of squares, with 3 df, to make specific comparisons among the interventions. One contrast (1 df) was used to compare the WL and GEM interventions. The F-test for a second contrast (2 df) was used to compare the three GEM versions regarding changes in HbA1c, followed by pairwise comparisons among GEM versions. χ2 tests were used to compare categorical outcomes among the interventions. Two-tailed probabilities were used to interpret all results. To address possible alpha error from multiple comparisons, the Benjamini-Hochberg procedure was employed. |
|  | 2.7 If N/PN/NI to 2.6: Was there potential for a substantial impact (on the result) of the failure to analyse participants in the group to which they were randomized? | | | NA |  |
|  | **Risk of bias judgement** | | | **Some concerns** |  |
| **Bias due to missing outcome data** | 3.1 Were data for this outcome available for all, or nearly all, participants randomized? | | | N | dropout rate is 11%, 4 in the EX group(n=40), and 15 in the CO group(6/39, 4/51, 5/48). |
|  | 3.2 If N/PN/NI to 3.1: Is there evidence that result was not biased by missing outcome data? | | | N |  |
|  | 3.3 If N/PN to 3.2: Could missingness in the outcome depend on its true value? | | | NI |  |
|  | 3.4 If Y/PY/NI to 3.3: Is it likely that missingness in the outcome depended on its true value? | | | NI |  |
|  | **Risk of bias judgement** | | | **High** |  |
| **Bias in measurement of the outcome** | 4.1 Was the method of measuring the outcome inappropriate? | | | N | The following week, all participants wore a blinded activity monitor to quantify baseline sedentary behavior (hours active) and overall activity (total steps). They were also interviewed by telephone on two workdays and one weekend day for the researcher to complete the Automated Self-Administered 24-hour (ASA24) dietary recall to quantify daily consumption of carbohydrates, fats and proteins. |
|  | 4.2 Could measurement or ascertainment of the outcome have differed between intervention groups? | | | N |  |
|  | 4.3 Were outcome assessors aware of the intervention received by study participants? | | | NI |  |
|  | 4.4 If Y/PY/NI to 4.3: Could assessment of the outcome have been influenced by knowledge of intervention received? | | | PY |  |
|  | 4.5 If Y/PY/NI to 4.4: Is it likely that assessment of the outcome was influenced by knowledge of intervention received? | | | PY |  |
|  | **Risk of bias judgement** | | | **Some concerns** |  |
| **Bias in selection of the reported result** | 5.1 Were the data that produced this result analysed in accordance with a pre-specified analysis plan that was finalized before unblinded outcome data were available for analysis? | | | Y |  |
|  | 5.2 ... multiple eligible outcome measurements (e.g. scales, definitions, time points) within the outcome domain? | | | N |  |
|  | 5.3 ... multiple eligible analyses of the data? | | | N |  |
|  | **Risk of bias judgement** | | | **Low** |  |
| **Overall bias** | **Risk of bias judgement** | | | **High** |  |
|  |  |  |  |  |  |
|  |  |  |  |  |  |
| **Unique ID** | Cox2 2020 | **Study ID** | doi: 10.1210/jendso/bvaa118 | **Assessor** |  |
| **Ref or Label** | J Endocr Soc. 2020 Aug 18;4(11):bvaa118. | **Aim** | assignment to intervention (the 'intention-to-treat' effect) |  |  |
| **Experimental** | CGM+glycemic excursion minimization | **Comparator** | Routine care | **Source** | Journal article(s) |
| **Outcome** | A blinded activity monitor; Automated Self- Administered 24- hour (ASA24) | **Results** |  | **Weight** | 1 |
| **Domain** | **Signalling question** | | | **Response** | **Comments** |
| **Bias arising from the randomization process** | 1.1 Was the allocation sequence random? | | | NI |  |
|  | 1.2 Was the allocation sequence concealed until participants were enrolled and assigned to interventions? | | | NI |  |
|  | 1.3 Did baseline differences between intervention groups suggest a problem with the randomization process? | | | N | The 2 groups did not significantly differ on any demographic variables. |
|  | **Risk of bias judgement** | | | **Some concerns** |  |
| **Bias due to deviations from intended interventions** | 2.1.Were participants aware of their assigned intervention during the trial? | | | PY |  |
|  | 2.2.Were carers and people delivering the interventions aware of participants' assigned intervention during the trial? | | | PY |  |
|  | 2.3. If Y/PY/NI to 2.1 or 2.2: Were there deviations from the intended intervention that arose because of the experimental context? | | | N |  |
|  | 2.4 If Y/PY to 2.3: Were these deviations likely to have affected the outcome? | | | NA |  |
|  | 2.5. If Y/PY/NI to 2.4: Were these deviations from intended intervention balanced between groups? | | | NA |  |
|  | 2.6 Was an appropriate analysis used to estimate the effect of assignment to intervention? | | | PY | Pre and post change scores for RC and GEMCGM were compared in analyses of covariance, with baseline measures serving as the covariant. SPSS version 25 was used to perform the analyses. |
|  | 2.7 If N/PN/NI to 2.6: Was there potential for a substantial impact (on the result) of the failure to analyse participants in the group to which they were randomized? | | | NA |  |
|  | **Risk of bias judgement** | | | **Low** |  |
| **Bias due to missing outcome data** | 3.1 Were data for this outcome available for all, or nearly all, participants randomized? | | | Y |  |
|  | 3.2 If N/PN/NI to 3.1: Is there evidence that result was not biased by missing outcome data? | | | NA |  |
|  | 3.3 If N/PN to 3.2: Could missingness in the outcome depend on its true value? | | | NA |  |
|  | 3.4 If Y/PY/NI to 3.3: Is it likely that missingness in the outcome depended on its true value? | | | NA |  |
|  | **Risk of bias judgement** | | | **Low** |  |
| **Bias in measurement of the outcome** | 4.1 Was the method of measuring the outcome inappropriate? | | | N | The following week participants wore a blinded activity monitor (Fitbit Charge 2), and were interviewed over the telephone on 2 work days and 1 weekend day to complete the automated self-administered 24-hour dietary recall dietary recall. |
|  | 4.2 Could measurement or ascertainment of the outcome have differed between intervention groups? | | | N |  |
|  | 4.3 Were outcome assessors aware of the intervention received by study participants? | | | NI |  |
|  | 4.4 If Y/PY/NI to 4.3: Could assessment of the outcome have been influenced by knowledge of intervention received? | | | PY |  |
|  | 4.5 If Y/PY/NI to 4.4: Is it likely that assessment of the outcome was influenced by knowledge of intervention received? | | | PY |  |
|  | **Risk of bias judgement** | | | **Some concerns** |  |
| **Bias in selection of the reported result** | 5.1 Were the data that produced this result analysed in accordance with a pre-specified analysis plan that was finalized before unblinded outcome data were available for analysis? | | | Y |  |
|  | 5.2 ... multiple eligible outcome measurements (e.g. scales, definitions, time points) within the outcome domain? | | | N |  |
|  | 5.3 ... multiple eligible analyses of the data? | | | N |  |
|  | **Risk of bias judgement** | | | **Low** |  |
| **Overall bias** | **Risk of bias judgement** | | | **Some concerns** |  |
|  |  |  |  |  |  |
|  |  |  |  |  |  |
| **Unique ID** | Cox 2021 | **Study ID** | DOI: 10.1136/bmjdrc-2021-002403 | **Assessor** |  |
| **Ref or Label** | BMJ Open Diabetes Res Care, 2021 Nov;9(2):e002403. | **Aim** | assignment to intervention (the 'intention-to-treat' effect) |  |  |
| **Experimental** | CGM+ group education on diet and PA. | **Comparator** | group education on diet and PA. | **Source** | Journal article(s) |
| **Outcome** | Carbohydrates Routinely Consumed (servings) | **Results** |  | **Weight** | 1 |
| **Domain** | **Signalling question** | | | **Response** | **Comments** |
| **Bias arising from the randomization process** | 1.1 Was the allocation sequence random? | | | NI | Trial registration number: NCT03196895 |
|  | 1.2 Was the allocation sequence concealed until participants were enrolled and assigned to interventions? | | | NI |  |
|  | 1.3 Did baseline differences between intervention groups suggest a problem with the randomization process? | | | N | Baseline variables for responders and non-responders in the GEM and WL groups, with contrasts (p value levels) |
|  | **Risk of bias judgement** | | | **Some concerns** |  |
| **Bias due to deviations from intended interventions** | 2.1.Were participants aware of their assigned intervention during the trial? | | | PY |  |
|  | 2.2.Were carers and people delivering the interventions aware of participants' assigned intervention during the trial? | | | PY |  |
|  | 2.3. If Y/PY/NI to 2.1 or 2.2: Were there deviations from the intended intervention that arose because of the experimental context? | | | N |  |
|  | 2.4 If Y/PY to 2.3: Were these deviations likely to have affected the outcome? | | | NA |  |
|  | 2.5. If Y/PY/NI to 2.4: Were these deviations from intended intervention balanced between groups? | | | NA |  |
|  | 2.6 Was an appropriate analysis used to estimate the effect of assignment to intervention? | | | Y | responders and no-responders analysis  The analyses included all available participants for each group and were like those done for the 3-month postassessment report.7 An analysis of covariance of pre to follow-up change scores, covarying baseline metrics, was conducted in SAS V.9.4. Orthogonal contrasts compared GEM to WL. The Benjamini-Hochberg procedure16 was employed to control for multiple comparisons, correcting for all p values in each table. P value levels with an asterisk (*) in tables indicate significant contrasts following this correction. Logistic regression analysis was employed to identify predictors of sustainability. Only those variables significant at post-treatment were entered into the regression model to predict responders at follow-up |
|  | 2.7 If N/PN/NI to 2.6: Was there potential for a substantial impact (on the result) of the failure to analyse participants in the group to which they were randomized? | | | NA |  |
|  | **Risk of bias judgement** | | | **Low** |  |
| **Bias due to missing outcome data** | 3.1 Were data for this outcome available for all, or nearly all, participants randomized? | | | PY |  |
|  | 3.2 If N/PN/NI to 3.1: Is there evidence that result was not biased by missing outcome data? | | | NA |  |
|  | 3.3 If N/PN to 3.2: Could missingness in the outcome depend on its true value? | | | NA |  |
|  | 3.4 If Y/PY/NI to 3.3: Is it likely that missingness in the outcome depended on its true value? | | | NA |  |
|  | **Risk of bias judgement** | | | **Low** |  |
| **Bias in measurement of the outcome** | 4.1 Was the method of measuring the outcome inappropriate? | | | NI | There is no specific introduction to dietary assessment methods in the main text. |
|  | 4.2 Could measurement or ascertainment of the outcome have differed between intervention groups? | | | NI |  |
|  | 4.3 Were outcome assessors aware of the intervention received by study participants? | | | NI |  |
|  | 4.4 If Y/PY/NI to 4.3: Could assessment of the outcome have been influenced by knowledge of intervention received? | | | PY |  |
|  | 4.5 If Y/PY/NI to 4.4: Is it likely that assessment of the outcome was influenced by knowledge of intervention received? | | | PY |  |
|  | **Risk of bias judgement** | | | **High** |  |
| **Bias in selection of the reported result** | 5.1 Were the data that produced this result analysed in accordance with a pre-specified analysis plan that was finalized before unblinded outcome data were available for analysis? | | | Y |  |
|  | 5.2 ... multiple eligible outcome measurements (e.g. scales, definitions, time points) within the outcome domain? | | | N |  |
|  | 5.3 ... multiple eligible analyses of the data? | | | N |  |
|  | **Risk of bias judgement** | | | **Low** |  |
| **Overall bias** | **Risk of bias judgement** | | | **High** |  |
|  |  |  |  |  |  |
|  |  |  |  |  |  |
| **Unique ID** | Kitazawa 2024 | **Study ID** | doi: 10.1210/clinem/dgad639 | **Assessor** |  |
| **Ref or Label** | J Clin Endocrinol Metab. 2023 Nov 1;109(4):1060–1070. | **Aim** | assignment to intervention (the 'intention-to-treat' effect) |  |  |
| **Experimental** | Unblinded CGM and Health 2Sync mobile app | **Comparator** | No lifestyle modification information and no use of health care related smartphone applications | **Source** | Journal article(s) |
| **Outcome** | Japanese version of the International Physical Activity Questionnaire short form; Brief Diet History Questionnaire | **Results** |  | **Weight** | 1 |
| **Domain** | **Signalling question** | | | **Response** | **Comments** |
| **Bias arising from the randomization process** | 1.1 Was the allocation sequence random? | | | Y | Study participants were randomly assigned (1:1) to either the smartphone app and isCGM intervention group or the control group on a 1:1 basis according to sex, age (≤45 years or >45 years), and preassignment HbA1c (≥6.0% or <6.0%) using the minimization method with a web-based electronic data capture system (UHCT ACReSS) provided by a third party (University Hospital Clinical Trial Alliance, Tokyo, Japan). Neither the researchers nor the participants were blinded. |
|  | 1.2 Was the allocation sequence concealed until participants were enrolled and assigned to interventions? | | | Y |  |
|  | 1.3 Did baseline differences between intervention groups suggest a problem with the randomization process? | | | PN | Table 1 shows the baseline characteristics and results of physical examinations, blood tests, blood glucose measurements with retrospective CGM, and questionnaires obtained 2 weeks before the study period. |
|  | **Risk of bias judgement** | | | **Low** |  |
| **Bias due to deviations from intended interventions** | 2.1.Were participants aware of their assigned intervention during the trial? | | | Y | Neither the researchers nor the participants were blinded. |
|  | 2.2.Were carers and people delivering the interventions aware of participants' assigned intervention during the trial? | | | Y |  |
|  | 2.3. If Y/PY/NI to 2.1 or 2.2: Were there deviations from the intended intervention that arose because of the experimental context? | | | NI |  |
|  | 2.4 If Y/PY to 2.3: Were these deviations likely to have affected the outcome? | | | NA |  |
|  | 2.5. If Y/PY/NI to 2.4: Were these deviations from intended intervention balanced between groups? | | | NA |  |
|  | 2.6 Was an appropriate analysis used to estimate the effect of assignment to intervention? | | | Y | full analysis set and per protocol set  Also, we compared the amount of change between the 2 groups for each endpoint using an analysis of covariance with each baseline characteristic as an adjustment variable. |
|  | 2.7 If N/PN/NI to 2.6: Was there potential for a substantial impact (on the result) of the failure to analyse participants in the group to which they were randomized? | | | NA |  |
|  | **Risk of bias judgement** | | | **Some concerns** |  |
| **Bias due to missing outcome data** | 3.1 Were data for this outcome available for all, or nearly all, participants randomized? | | | Y |  |
|  | 3.2 If N/PN/NI to 3.1: Is there evidence that result was not biased by missing outcome data? | | | NA |  |
|  | 3.3 If N/PN to 3.2: Could missingness in the outcome depend on its true value? | | | NA |  |
|  | 3.4 If Y/PY/NI to 3.3: Is it likely that missingness in the outcome depended on its true value? | | | NA |  |
|  | **Risk of bias judgement** | | | **Low** |  |
| **Bias in measurement of the outcome** | 4.1 Was the method of measuring the outcome inappropriate? | | | N | Health2Sync app usage was evaluated by the number of days the app was opened between days 1 and 14, 15 and 28, 29 and 42, 43 and 56, 57 and 70, and 71 and 84 and the number of food logs and the number of physical activity logs. |
|  | 4.2 Could measurement or ascertainment of the outcome have differed between intervention groups? | | | N |  |
|  | 4.3 Were outcome assessors aware of the intervention received by study participants? | | | PY | Neither the researchers nor the participants were blinded. |
|  | 4.4 If Y/PY/NI to 4.3: Could assessment of the outcome have been influenced by knowledge of intervention received? | | | PY |  |
|  | 4.5 If Y/PY/NI to 4.4: Is it likely that assessment of the outcome was influenced by knowledge of intervention received? | | | PY |  |
|  | **Risk of bias judgement** | | | **High** |  |
| **Bias in selection of the reported result** | 5.1 Were the data that produced this result analysed in accordance with a pre-specified analysis plan that was finalized before unblinded outcome data were available for analysis? | | | Y |  |
|  | 5.2 ... multiple eligible outcome measurements (e.g. scales, definitions, time points) within the outcome domain? | | | N |  |
|  | 5.3 ... multiple eligible analyses of the data? | | | N |  |
|  | **Risk of bias judgement** | | | **Low** |  |
| **Overall bias** | **Risk of bias judgement** | | | **High** |  |
|  |  |  |  |  |  |
|  |  |  |  |  |  |
| **Unique ID** | Kyto 2024 | **Study ID** | https://doi.org/10.1016/j.ajog.2024.02.303 | **Assessor** |  |
| **Ref or Label** | Am J Obstet Gynecol, 2024 Nov;231(5):541.e1-541.e16. | **Aim** | assignment to intervention (the 'intention-to-treat' effect) |  |  |
| **Experimental** | CGM with standard care + mobile application with wearable sensors | **Comparator** | standard care | **Source** | Journal article(s) |
| **Outcome** | A blind accelerometer ; Semiquantitative 142-item food-frequency questionnaire | **Results** |  | **Weight** | 1 |
| **Domain** | **Signalling question** | | | **Response** | **Comments** |
| **Bias arising from the randomization process** | 1.1 Was the allocation sequence random? | | | PY | We employed randomly permuted blocks that were stratified by gestational weeks of the GDM diagnosis (early, <24 weeks or late, >24–28 weeks), parity (primiparous or multiparous), and body mass index (<30 or ≥30 kg/m2). The randomization into the intervention and the control arm was performed by study nurses who dispended the next sequentially numbered subject code and opened the corresponding opaque and sealed envelope, which included the arm to be assigned to the subject. The study nurses were masked to the results until the end of study. |
|  | 1.2 Was the allocation sequence concealed until participants were enrolled and assigned to interventions? | | | PY |  |
|  | 1.3 Did baseline differences between intervention groups suggest a problem with the randomization process? | | | N | At the baseline, no between-arm differences were found except for protein intake (difference, −1.2%; P=.01) (Table 1). |
|  | **Risk of bias judgement** | | | **Low** |  |
| **Bias due to deviations from intended interventions** | 2.1.Were participants aware of their assigned intervention during the trial? | | | PY | The study nurses were masked to the results until the end of study. |
|  | 2.2.Were carers and people delivering the interventions aware of participants' assigned intervention during the trial? | | | N |  |
|  | 2.3. If Y/PY/NI to 2.1 or 2.2: Were there deviations from the intended intervention that arose because of the experimental context? | | | PN |  |
|  | 2.4 If Y/PY to 2.3: Were these deviations likely to have affected the outcome? | | | NA |  |
|  | 2.5. If Y/PY/NI to 2.4: Were these deviations from intended intervention balanced between groups? | | | NA |  |
|  | 2.6 Was an appropriate analysis used to estimate the effect of assignment to intervention? | | | Y | We performed the analyses according to the intention-to-treat principle; all the pregnancy data, including glucose, physical activity, diet, etc., were considered until delivery or drop-out, along with delivery and neonatal data for every participant. A 2-sided t test was conducted for the continuous outcomes with a significance level of 5%. For the binary variables, we used a chi-square test or Fisher exact test when the expected value was <10. Concerning eMOM use, we investigated the linear correlation (Pearson) between the number of interactions with eMOM and continuous outcomes. For the binary outcomes, we employed a logistic regression. A more detailed description is available in Supplemental Appendix. |
|  | 2.7 If N/PN/NI to 2.6: Was there potential for a substantial impact (on the result) of the failure to analyse participants in the group to which they were randomized? | | | NA |  |
|  | **Risk of bias judgement** | | | **Low** |  |
| **Bias due to missing outcome data** | 3.1 Were data for this outcome available for all, or nearly all, participants randomized? | | | Y |  |
|  | 3.2 If N/PN/NI to 3.1: Is there evidence that result was not biased by missing outcome data? | | | NA |  |
|  | 3.3 If N/PN to 3.2: Could missingness in the outcome depend on its true value? | | | NA |  |
|  | 3.4 If Y/PY/NI to 3.3: Is it likely that missingness in the outcome depended on its true value? | | | NA |  |
|  | **Risk of bias judgement** | | | **Low** |  |
| **Bias in measurement of the outcome** | 4.1 Was the method of measuring the outcome inappropriate? | | | N | In the preceding month, the diet data were collected from both arms using a digital, semiquantitative 142-item food-frequency questionnaire during study visits. The dietary intake data in the intervention arm were also collected from a 3-day food diary (part of eMOM).    During study visits, physical activity data were obtained from both arms with a blind accelerometer (RM42, UKK-Institute, Finland) that classified physical activity into sedentary behavior, light physical activity, and moderate to vigorous physical activity. For the intervention arm, physical activity data were also collected with the activity tracker (Garmin). |
|  | 4.2 Could measurement or ascertainment of the outcome have differed between intervention groups? | | | N |  |
|  | 4.3 Were outcome assessors aware of the intervention received by study participants? | | | NI |  |
|  | 4.4 If Y/PY/NI to 4.3: Could assessment of the outcome have been influenced by knowledge of intervention received? | | | PY |  |
|  | 4.5 If Y/PY/NI to 4.4: Is it likely that assessment of the outcome was influenced by knowledge of intervention received? | | | PY |  |
|  | **Risk of bias judgement** | | | **Some concerns** |  |
| **Bias in selection of the reported result** | 5.1 Were the data that produced this result analysed in accordance with a pre-specified analysis plan that was finalized before unblinded outcome data were available for analysis? | | | Y |  |
|  | 5.2 ... multiple eligible outcome measurements (e.g. scales, definitions, time points) within the outcome domain? | | | N |  |
|  | 5.3 ... multiple eligible analyses of the data? | | | N |  |
|  | **Risk of bias judgement** | | | **Low** |  |
| **Overall bias** | **Risk of bias judgement** | | | **Low** |  |
|  |  |  |  |  |  |
|  |  |  |  |  |  |
| **Unique ID** | Lee 2022 | **Study ID** | DOI: 10.3389/fendo.2022.1054697 | **Assessor** |  |
| **Ref or Label** | Front Endocrinol (Lausanne), 2022 Nov 25:13:1054697. | **Aim** | assignment to intervention (the 'intention-to-treat' effect) |  |  |
| **Experimental** | Unblinded CGM and retrospective CGM-based individual guidance | **Comparator** | Unblinded CGM without intervention | **Source** | Journal article(s) |
| **Outcome** | Hours of exercise per week; Number of meals per day | **Results** |  | **Weight** | 1 |
| **Domain** | **Signalling question** | | | **Response** | **Comments** |
| **Bias arising from the randomization process** | 1.1 Was the allocation sequence random? | | | NI | The participants were randomly assigned in a 1:1 ratio to either the intervention group or the control group. The intervention group used FGM for 12 weeks with remote intervention by medical staff and the control group used FGM without intervention. The participants were stratified at randomization according to their baseline HbA1c level (<9.0% or ≥9.0%) and age (<47 or ≥47 years). |
|  | 1.2 Was the allocation sequence concealed until participants were enrolled and assigned to interventions? | | | NI |  |
|  | 1.3 Did baseline differences between intervention groups suggest a problem with the randomization process? | | | N | The baseline characteristics of participants are shown in Table 1. There were no significant differences in baseline demographics and clinical characteristics between the two groups. |
|  | **Risk of bias judgement** | | | **Some concerns** |  |
| **Bias due to deviations from intended interventions** | 2.1.Were participants aware of their assigned intervention during the trial? | | | PY | Participants underwent different procedures that cannot be blinded. |
|  | 2.2.Were carers and people delivering the interventions aware of participants' assigned intervention during the trial? | | | PY |  |
|  | 2.3. If Y/PY/NI to 2.1 or 2.2: Were there deviations from the intended intervention that arose because of the experimental context? | | | NI |  |
|  | 2.4 If Y/PY to 2.3: Were these deviations likely to have affected the outcome? | | | NA |  |
|  | 2.5. If Y/PY/NI to 2.4: Were these deviations from intended intervention balanced between groups? | | | NA |  |
|  | 2.6 Was an appropriate analysis used to estimate the effect of assignment to intervention? | | | N | A total of 34 participants (94%) with 17 in each group completed the study and were analyzed for the per-protocol population. |
|  | 2.7 If N/PN/NI to 2.6: Was there potential for a substantial impact (on the result) of the failure to analyse participants in the group to which they were randomized? | | | PY |  |
|  | **Risk of bias judgement** | | | **High** |  |
| **Bias due to missing outcome data** | 3.1 Were data for this outcome available for all, or nearly all, participants randomized? | | | Y |  |
|  | 3.2 If N/PN/NI to 3.1: Is there evidence that result was not biased by missing outcome data? | | | NA |  |
|  | 3.3 If N/PN to 3.2: Could missingness in the outcome depend on its true value? | | | NA |  |
|  | 3.4 If Y/PY/NI to 3.3: Is it likely that missingness in the outcome depended on its true value? | | | NA |  |
|  | **Risk of bias judgement** | | | **Low** |  |
| **Bias in measurement of the outcome** | 4.1 Was the method of measuring the outcome inappropriate? | | | N | In addition, changes in the psychosocial, behavioral, and physical variables were assessed as outcomes; i.e., scores of the DTSQs, DTSQc, PHQ-9, and GAD-7 questionnaires, total daily doses of insulin, the number of scans per day, lifestyle factors such as diet and exercise, and anthropometric variables. |
|  | 4.2 Could measurement or ascertainment of the outcome have differed between intervention groups? | | | N |  |
|  | 4.3 Were outcome assessors aware of the intervention received by study participants? | | | NI |  |
|  | 4.4 If Y/PY/NI to 4.3: Could assessment of the outcome have been influenced by knowledge of intervention received? | | | PY |  |
|  | 4.5 If Y/PY/NI to 4.4: Is it likely that assessment of the outcome was influenced by knowledge of intervention received? | | | PY |  |
|  | **Risk of bias judgement** | | | **Some concerns** |  |
| **Bias in selection of the reported result** | 5.1 Were the data that produced this result analysed in accordance with a pre-specified analysis plan that was finalized before unblinded outcome data were available for analysis? | | | Y |  |
|  | 5.2 ... multiple eligible outcome measurements (e.g. scales, definitions, time points) within the outcome domain? | | | N |  |
|  | 5.3 ... multiple eligible analyses of the data? | | | N |  |
|  | **Risk of bias judgement** | | | **Low** |  |
| **Overall bias** | **Risk of bias judgement** | | | **High** |  |
|  |  |  |  |  |  |
|  |  |  |  |  |  |
| **Unique ID** | Yan 2022 | **Study ID** | doi: 10.3389/fendo.2022.832102 | **Assessor** |  |
| **Ref or Label** | Front Endocrinol (Lausanne), 2022 Feb 10:13:832102. | **Aim** | assignment to intervention (the 'intention-to-treat' effect) |  |  |
| **Experimental** | Unblinded CGM and CGM-based guidance | **Comparator** | Blinded CGM (with feedback after wear) | **Source** | Journal article(s) |
| **Outcome** | Daily exercise time(min/day); Energy intake, number of meals. | **Results** |  | **Weight** | 1 |
| **Domain** | **Signalling question** | | | **Response** | **Comments** |
| **Bias arising from the randomization process** | 1.1 Was the allocation sequence random? | | | NI |  |
|  | 1.2 Was the allocation sequence concealed until participants were enrolled and assigned to interventions? | | | NI |  |
|  | 1.3 Did baseline differences between intervention groups suggest a problem with the randomization process? | | | N | Participant characteristics at baseline were similar between the study groups except insulin dose and the percentage of acarbose use |
|  | **Risk of bias judgement** | | | **Some concerns** |  |
| **Bias due to deviations from intended interventions** | 2.1.Were participants aware of their assigned intervention during the trial? | | | Y |  |
|  | 2.2.Were carers and people delivering the interventions aware of participants' assigned intervention during the trial? | | | Y |  |
|  | 2.3. If Y/PY/NI to 2.1 or 2.2: Were there deviations from the intended intervention that arose because of the experimental context? | | | NI |  |
|  | 2.4 If Y/PY to 2.3: Were these deviations likely to have affected the outcome? | | | NA |  |
|  | 2.5. If Y/PY/NI to 2.4: Were these deviations from intended intervention balanced between groups? | | | NA |  |
|  | 2.6 Was an appropriate analysis used to estimate the effect of assignment to intervention? | | | N | All statistical analyses were performed using SPSS version 22.0 software (IBM Corp., Foster City, CA, USA). All variables were tested for normal distribution. Data are presented as mean (95% CI) or percentage. Differences between the two groups were examined using Student’s unpaired t-test (insulin dose) or the Mann–Whitney U-test (age, diabetic duration, BMI, exercise time, carbohydrate, calories, and daily meal frequency at baseline). The parameters (TIR, MBG, CV, and SDBG) assessed by three FGMs, and HbA1c, C-peptide, insulin, and metformin dose, and lifestyles at baseline and endpoint were analyzed by a mixed-model ANOVA with time as the within-subject factor and groups as the between-subject factor. The categorical data were examined with the chi-square test. All comparisons were 2-sided at a 5% significance level. A p value < 0.05 was considered statistically significant. |
|  | 2.7 If N/PN/NI to 2.6: Was there potential for a substantial impact (on the result) of the failure to analyse participants in the group to which they were randomized? | | | PY | The experimental group and control group had 101 and 102 individuals, respectively, with 83 and 89 individuals included in the final analysis. |
|  | **Risk of bias judgement** | | | **High** |  |
| **Bias due to missing outcome data** | 3.1 Were data for this outcome available for all, or nearly all, participants randomized? | | | N | dropoutout rate is 15%. |
|  | 3.2 If N/PN/NI to 3.1: Is there evidence that result was not biased by missing outcome data? | | | N |  |
|  | 3.3 If N/PN to 3.2: Could missingness in the outcome depend on its true value? | | | NI |  |
|  | 3.4 If Y/PY/NI to 3.3: Is it likely that missingness in the outcome depended on its true value? | | | NI |  |
|  | **Risk of bias judgement** | | | **High** |  |
| **Bias in measurement of the outcome** | 4.1 Was the method of measuring the outcome inappropriate? | | | N | Secondary outcomes included TBR, percentage time spent in hyperglycemia > 10.0 mmol/l (time above target range, TAR), 24 h mean blood glucose (MBG), standard deviation of blood glucose (SDBG), CV, hourly mean blood glucose (the average of 14 days during FGM), HbA1c, C-peptide, and daily exercise time, energy intake, number of meals, and insulin dose per day during FGM. |
|  | 4.2 Could measurement or ascertainment of the outcome have differed between intervention groups? | | | N |  |
|  | 4.3 Were outcome assessors aware of the intervention received by study participants? | | | NI |  |
|  | 4.4 If Y/PY/NI to 4.3: Could assessment of the outcome have been influenced by knowledge of intervention received? | | | PY |  |
|  | 4.5 If Y/PY/NI to 4.4: Is it likely that assessment of the outcome was influenced by knowledge of intervention received? | | | PY |  |
|  | **Risk of bias judgement** | | | **Some concerns** |  |
| **Bias in selection of the reported result** | 5.1 Were the data that produced this result analysed in accordance with a pre-specified analysis plan that was finalized before unblinded outcome data were available for analysis? | | | Y |  |
|  | 5.2 ... multiple eligible outcome measurements (e.g. scales, definitions, time points) within the outcome domain? | | | N |  |
|  | 5.3 ... multiple eligible analyses of the data? | | | N |  |
|  | **Risk of bias judgement** | | | **Low** |  |
| **Overall bias** | **Risk of bias judgement** | | | **High** |  |
|  |  |  |  |  |  |
|  |  |  |  |  |  |
| **Unique ID** | Yoo 2008 | **Study ID** | doi: 10.1016/j.diabres.2008.06.015 | **Assessor** |  |
| **Ref or Label** | Diabetes Res Clin Pract, 2008 Oct;82(1):73-9. | **Aim** | assignment to intervention (the 'intention-to-treat' effect) |  |  |
| **Experimental** | Unblinded CGM and CGM-based guidance | **Comparator** | Individual education and glucometer-based advice. | **Source** | Journal article(s) |
| **Outcome** | Exercise time per week (min/week); 3-day food records analyzed by Can-Pro 3.0 | **Results** |  | **Weight** | 1 |
| **Domain** | **Signalling question** | | | **Response** | **Comments** |
| **Bias arising from the randomization process** | 1.1 Was the allocation sequence random? | | | Y | In the study, we successfully recruited 65 patients. These subjects were randomly assigned to the Guardian RT (i.e., RT-CGM) or SMBG groups by a random number table (Fig. 1). The allocation process was concealed so the participants were unable to influence one another. |
|  | 1.2 Was the allocation sequence concealed until participants were enrolled and assigned to interventions? | | | Y |  |
|  | 1.3 Did baseline differences between intervention groups suggest a problem with the randomization process? | | | N | There were no significant differences of baseline characteristics between the two groups. |
|  | **Risk of bias judgement** | | | **Low** |  |
| **Bias due to deviations from intended interventions** | 2.1.Were participants aware of their assigned intervention during the trial? | | | PY | In the study, we successfully recruited 65 patients. These subjects were randomly assigned to the Guardian RT (i.e., RT-CGM) or SMBG groups by a random number table (Fig. 1). The allocation process was concealed so the participants were unable to influence one another. |
|  | 2.2.Were carers and people delivering the interventions aware of participants' assigned intervention during the trial? | | | PY |  |
|  | 2.3. If Y/PY/NI to 2.1 or 2.2: Were there deviations from the intended intervention that arose because of the experimental context? | | | N |  |
|  | 2.4 If Y/PY to 2.3: Were these deviations likely to have affected the outcome? | | | NA |  |
|  | 2.5. If Y/PY/NI to 2.4: Were these deviations from intended intervention balanced between groups? | | | NA |  |
|  | 2.6 Was an appropriate analysis used to estimate the effect of assignment to intervention? | | | N | For demographic and baseline characteristics, continuous and categorical variables were summarized as the means ± standard deviation (S.D.) and frequency with percentile, respectively, in each group. Differences in demographic and baseline variables between the Guardian RT and SMBG groups were tested using the Wilcoxon rank sum test for continuous variables and the Fisher's exact test for categorical variables. To compare mean changes in anthropometric, biochemical variables, and life style habits between the Guardian RT and SMBG groups over time, repeated measures ANOVA was used. All statistical analyses were performed using SPSS for Windows (version 10.0; SPSS, Inc., Chicago, IL, USA). |
|  | 2.7 If N/PN/NI to 2.6: Was there potential for a substantial impact (on the result) of the failure to analyse participants in the group to which they were randomized? | | | PN | There were 32 people in the experimental group and 33 people in the control group, and 29 people and 28 people were finally included in the analysis. |
|  | **Risk of bias judgement** | | | **Some concerns** |  |
| **Bias due to missing outcome data** | 3.1 Were data for this outcome available for all, or nearly all, participants randomized? | | | N | Dropout rate is 12%. |
|  | 3.2 If N/PN/NI to 3.1: Is there evidence that result was not biased by missing outcome data? | | | N |  |
|  | 3.3 If N/PN to 3.2: Could missingness in the outcome depend on its true value? | | | NI |  |
|  | 3.4 If Y/PY/NI to 3.3: Is it likely that missingness in the outcome depended on its true value? | | | NI |  |
|  | **Risk of bias judgement** | | | **High** |  |
| **Bias in measurement of the outcome** | 4.1 Was the method of measuring the outcome inappropriate? | | | N | The secondary end point was the difference in the change in diet and exercise habits between the Guardian RT and SMBG groups. To assess the change in the patients’ diet and exercise habits, participants in both groups maintained diaries covering 3 days of meals and 7 days of physical activity at baseline and 3 months later. The 3-day food records were computed by one nutritionist and analyzed by Can-Pro 3.0 [11], a computerized nutrient intake assessment software program developed by the Korean Nutrition Society. Physical activity was recorded in time (min) spent for exercise during 1 week. |
|  | 4.2 Could measurement or ascertainment of the outcome have differed between intervention groups? | | | N |  |
|  | 4.3 Were outcome assessors aware of the intervention received by study participants? | | | NI |  |
|  | 4.4 If Y/PY/NI to 4.3: Could assessment of the outcome have been influenced by knowledge of intervention received? | | | PY |  |
|  | 4.5 If Y/PY/NI to 4.4: Is it likely that assessment of the outcome was influenced by knowledge of intervention received? | | | PY |  |
|  | **Risk of bias judgement** | | | **Some concerns** |  |
| **Bias in selection of the reported result** | 5.1 Were the data that produced this result analysed in accordance with a pre-specified analysis plan that was finalized before unblinded outcome data were available for analysis? | | | Y |  |
|  | 5.2 ... multiple eligible outcome measurements (e.g. scales, definitions, time points) within the outcome domain? | | | N |  |
|  | 5.3 ... multiple eligible analyses of the data? | | | N |  |
|  | **Risk of bias judgement** | | | **Low** |  |
| **Overall bias** | **Risk of bias judgement** | | | **High** |  |
